# Molecular oxygen enhances H_2_O_2_ utilization for the photocatalytic conversion of methane to liquid-phase oxygenates

**DOI:** 10.1038/s41467-022-34563-4

**Published:** 2022-11-05

**Authors:** Xiao Sun, Xuanye Chen, Cong Fu, Qingbo Yu, Xu-Sheng Zheng, Fei Fang, Yuanxu Liu, Junfa Zhu, Wenhua Zhang, Weixin Huang

**Affiliations:** 1 Hefei National Research Center for Physical Sciences at the Microscale, iChEM, Key Laboratory of Surface and Interface Chemistry and Energy Catalysis of Anhui Higher Education Institutes, School of Chemistry and Materials Science, University of Science and Technology of China, 230026 Hefei, China; 2 Department of Materials Science and Engineering, Anhui University of Science and Technology, 232001 Huainan, China; 3 National Synchrotron Radiation Laboratory, University of Science and Technology of China, Hefei, 230029 Anhui China; 4 School of Pharmacy, Anhui University of Chinese Medicine, Anhui Academy of Chinese Medicine, Hefei, 230012 Anhui China; 5 Dalian National Laboratory for Clean Energy, Chinese Academy of Sciences, 116023 Dalian, China

**Keywords:** Photocatalysis, Catalytic mechanisms, Environmental chemistry

## Abstract

H_2_O_2_ is widely used as an oxidant for photocatalytic methane conversion to value-added chemicals over oxide-based photocatalysts under mild conditions, but suffers from low utilization efficiencies. Herein, we report that O_2_ is an efficient molecular additive to enhance the utilization efficiency of H_2_O_2_ by suppressing H_2_O_2_ adsorption on oxides and consequent photogenerated holes-mediated H_2_O_2_ dissociation into O_2_. In photocatalytic methane conversion over an anatase TiO_2_ nanocrystals predominantly enclosed by the {001} facets (denoted as TiO_2_{001})-C_3_N_4_ composite photocatalyst at room temperature and ambient pressure, O_2_ additive significantly enhances the utilization efficiency of H_2_O_2_ up to 93.3%, giving formic acid and liquid-phase oxygenates selectivities respectively of 69.8% and 97% and a formic acid yield of 486 μmol_HCOOH_·g_catalyst_^−1^·h^−1^. Efficient charge separation within TiO_2_{001}-C_3_N_4_ heterojunctions, photogenerated holes-mediated activation of CH_4_ into ·CH_3_ radicals on TiO_2_{001} and photogenerated electrons-mediated activation of H_2_O_2_ into ·OOH radicals on C_3_N_4_, and preferential dissociative adsorption of methanol on TiO_2_{001} are responsible for the active and selective photocatalytic conversion of methane to formic acid over TiO_2_{001}-C_3_N_4_ composite photocatalyst.

## Abstract (web summary)

The oxidation of methane to formic acid or related oxygenates relies on efficient reaction with H_2_O_2_. Here, the authors report a TiO_2_-based catalyst to selectively form formic acid by using molecular O_2_ additives to avoid unwanted side reactions.

## Introduction

Methane has been considered as an abundant and promising feedstock for future energy and chemical productions, especially after discovery of large reserves of shale gas and methane hydrate^1,2^. Direct conversion of methane to value-added chemicals has been attracting great interest, however, due to a stable C–H bond, a small polarizability, a high ionization potential and a low electron affinity stability of methane, it remains as a long-standing challenge^3–5^. Harsh reaction conditions, such as high temperatures^6–8^ and/or high pressures^9–15^, are required for traditional heterogeneous thermocatalytic selective conversion of methane. Recently, photocatalysis has been explored for selectively converting methane mainly to valuable liquid oxygenates at room temperature and ambient pressure^16–24^.

H_2_O_2_ is widely used as an oxidant for photocatalytic selective conversion of methane over oxide-based photocatalysts. Photocatalytic activation of H_2_O_2_ by photo-generated electrons into ·OH radicals (0.06 eV vs *RHE*)^25^ or ·OOH radicals (−0.38 eV vs *RHE*)^26^, depending on the conduction band edges of semiconductor photocatalysts, is generally considered as the key step. However, photocatalytic activation of H_2_O_2_ by photo-generated holes into O_2_ usually occurs facilely^25^, which strongly competes and decreases the utilization efficiency of H_2_O_2_ for the methane conversion, defined as the ratio of the H_2_O_2_ amount consumed for methane conversion against the total consumed H_2_O_2_ amount. So far, the highest utilization efficiency of H_2_O_2_, in the means of ·OH radicals, was reported as 72.3% in photocatalytic CH_4_ conversion over a Fenton-type Fe-based catalyst^21^. Adsorption of H_2_O_2_ molecules on photocatalyst surfaces is a prerequisite for occurrences of photocatalytic reactions. Here, we show O_2_ additive as a general strategy to enhance utilization efficiencies of H_2_O_2_ for the photocatalytic CH_4_ conversion over oxide-based photocatalysts up to 93.3% by suppressing the H_2_O_2_ adsorption on photocatalyst surfaces and the consequent side reaction of photocatalytic H_2_O_2_ dissociation into O_2_.

## Results

### Synthesis and structural characterizations

**Fig. 1:**
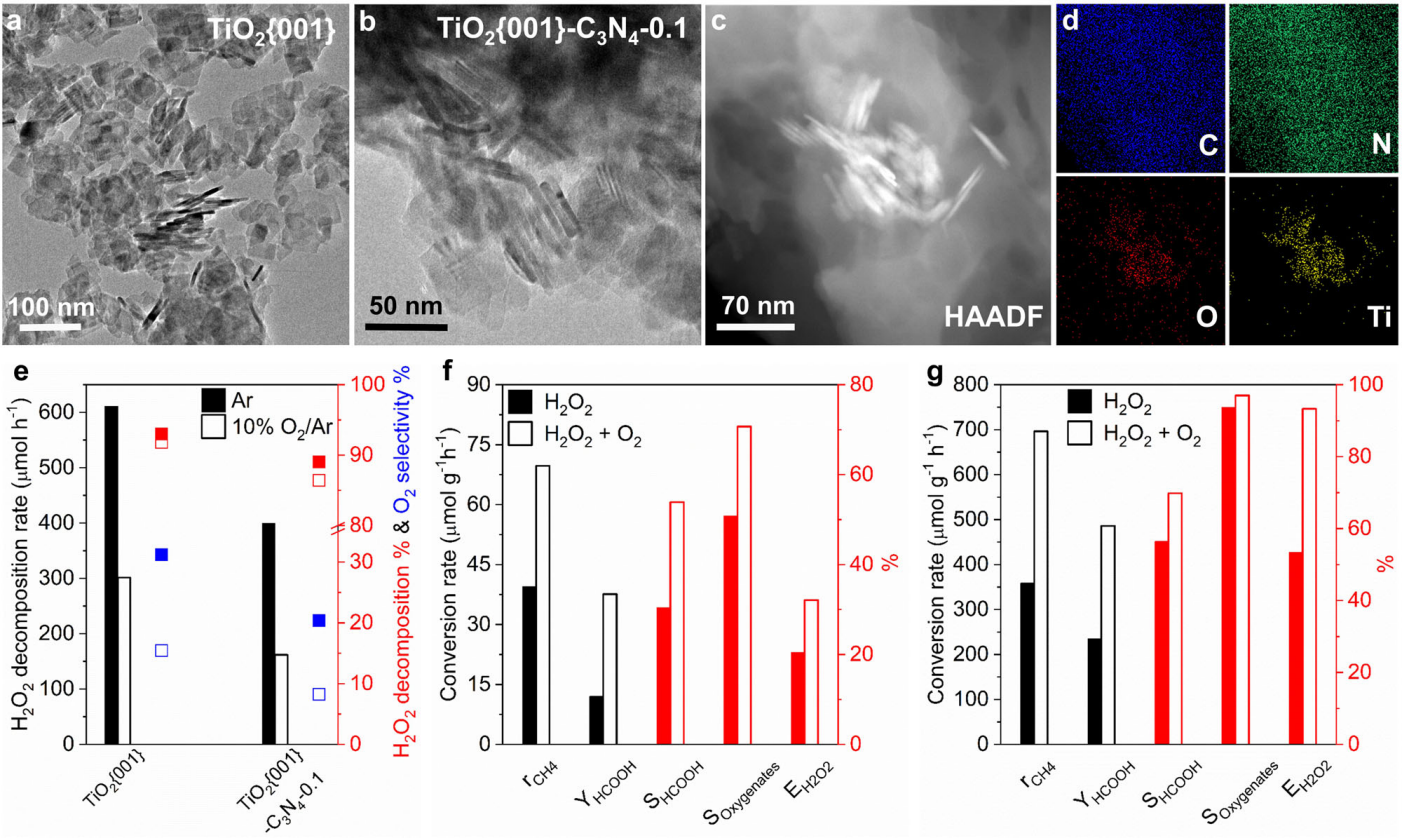
Photocatalysts and photocatalytic performance. **a** TEM image of TiO_2_{001}. **b** TEM, (**c**) HAADF and (**d**) element mapping images of TiO_2_{001}-C_3_N_4_−0.1. **e** H_2_O_2_ decomposition rate, H_2_O_2_ decomposition and O_2_ selectivity of photocatalytic H_2_O_2_ decomposition over TiO_2_{001} and TiO_2_{001}-C_3_N_4_−0.1 under the reaction condition of 165 μL H_2_O_2_ + 20 mL H_2_O in Ar or 10%O_2_/Ar. CH_4_ conversion rate (r_CH4_), yield(Y_HCOOH_) and selectivity (S_HCOOH_) of formic acid, selectivity of oxygenates (S_Oxygenates_), and H_2_O_2_ utilization efficiency (E_H2O2_) of photocatalytic CH_4_ conversion over (**f**) 20 mg TiO_2_{001} under the reaction condition of 8%CH_4_ + 92%Ar + 110 μL H_2_O_2_ + 20 mL H_2_O or 8%CH_4_ + 1.6%O_2_ + 90.4%Ar + 110 μL H_2_O_2_ + 20 mL H_2_O for 5 h and over (**g**) 20 mg TiO_2_{001}-C_3_N_4_−0.1 under the reaction condition of 8%CH_4_ + 92%Ar + 165 μL H_2_O_2_ + 20 mL H_2_O or 8%CH_4_ + 4%O_2_ + 88%Ar + 165 μL H_2_O_2_ + 20 mL H_2_O for 8 h at 298 K. Source data are provided as a Source Data file.

Anatase TiO_2_ nanocrystals (NCs) predominantly enclosed by the {001} facets (denoted as TiO_2_{001}), the {100} facets (denoted as TiO_2_{100}) and the {101} facets (denoted as TiO_2_{101}) were prepared following well-established recipes^27^. XRD patterns, TEM and HRETM images of as-synthesized various TiO_2_ NCs (Fig. 1a, Supplementary Fig. 1) agree with those reported previously^27^. TiO_2_ NCs-C_3_N_4_ composites were prepared by calcination of mixture of calculated amounts of dicyandiamide (C_2_H_4_N_4_) and TiO_2_ NCs in Ar at 550 °C and denoted as TiO_2_ NCs-C_3_N_4_-x, in which x was the actual TiO_2_:C_3_N_4_ mole ratio acquired by TGA analysis (Supplementary Fig. 2 and Table 1). TEM, HRTEM and element mapping images (Fig. 1b–d, Supplementary Fig. 3a–c) show that various TiO_2_ NCs preserve their original morphologies and form smooth anatase TiO_2_-g-C_3_N_4_ interfaces. We failed to observe clear lattice fringes of g-C_3_N_4_ in the HRTEM images (Supplementary Fig. 3d) likely due to the strong damage effect of high-energy electron beam on the structure of g-C_3_N_4_, but its presence in the TiO_2_ NCs-C_3_N_4_ composites is identified by XRD patterns (Supplementary Fig. 3e) and XPS spectra (Supplementary Fig. 3f).

### Photocatalytic performance

H_2_O_2_ barely decomposes at 300 K over various oxides (P25, ZnO, Fe_2_O_3_, WO_3_, CuO and V_2_O_5_) without Xe light illumination. Under Xe light illumination, H_2_O_2_ decomposition predominantly to O_2_ occurs slightly in an Ar atmosphere without the presence of oxides but substantially with the presence of oxides (Supplementary Table 2), demonstrating facile occurrence of photogenerated holes-mediated H_2_O_2_ decomposition to O_2_. Photocatalytic H_2_O_2_ decomposition over various TiO_2_ NCs was observed dependent on the surface structure. TiO_2_{001} NCs exhibit the lowest photocatalytic activity and O_2_ selectivity while TiO_2_{101} NCs exhibit the highest (Supplementary Table 3). C_3_N_4_ is poor in photocatalytic H_2_O_2_ decomposition, and comparing corresponding TiO_2_ NCs, TiO_2_ NCs-C_3_N_4_ composites exhibit much decreased photocatalytic activity and O_2_ selectivity (Supplementary Table 3). Interestingly, we found that photocatalytic H_2_O_2_ decomposition over oxides gets greatly suppressed in an O_2_/Ar atmosphere, together with slight decrease of O_2_ selectivity; moreover, such an O_2_ suppress effect varies with the structures of TiO_2_ NCs and TiO_2_ NCs-C_3_N_4_ composites (Supplementary Tables 2 and 3). As shown in Fig. 1e, the H_2_O_2_ decomposition percentage/H_2_O_2_ decomposition rate/O_2_ selectivity are 31.2%/610.9 μmol h^−1^/93.0% over TiO_2_{001} NCs in the Ar atmosphere and decrease to 15.4%/301.5 μmol h^−1^/91.8% in the 10% O_2_/Ar atmosphere, while they are 20.4%/399.4 μmol h^−1^/89.0% over TiO_2_{001}-C_3_N_4_−0.1 in the Ar atmosphere and decrease to 8.26%/161.7 μmol·h^−1^/86.4% in the 10% O_2_/Ar atmosphere.

The suppress effect of O_2_ on photocatalytic H_2_O_2_ decomposition into O_2_ was observed to generally enhance not only H_2_O_2_ utilization efficiency but also H_2_O_2_ conversion, and consequently CH_4_ conversion in aqueous-phase photocatalytic conversion of methane with H_2_O_2_ using oxide photocatalysts due to the reaction coupling between photocatalytic H_2_O_2_ and CH_4_ reactions (Supplementary Table 4). Under the studied condition, the H_2_O_2_ utilization efficiency and CH_4_ conversion with an O_2_ addition are 1.30–1.78 and 1.4–2.0 times of those without O_2_ addition, respectively. We then optimized the O_2_ enhancement effect and photocatalytic performance over TiO_2_ NCs and TiO_2_ NCs-C_3_N_4_ composites (Supplementary Tables 5–10), both of which were observed to vary with structures of TiO_2_ NCs. TiO_2_{001} NCs are more photocatalytic active than TiO_2_{100} and TiO_2_{101} NCs, and the produced liquid-phase oxygenates are CH_3_OH and HCOOH over TiO_2_{001} NCs and CH_3_OH over TiO_2_{100} and TiO_2_{101} NCs. Over TiO_2_{001} NCs (Fig. 1f), the O_2_ addition increases the methane conversion rate from 39.5 to 69.7 μmol·g_catalyst_^−1^·h^−1^, the selectivity of liquid-phase oxygenates and HCOOH respectively from 50.8% to 70.7% and from 30.4% to 53.9%, the HCOOH yield from 12.0 to 37.6 μmol·g_catalyst_^−1^ h^−1^, and the H_2_O_2_ utilization efficiency from 21.4% to 32.1%. TiO_2_ NCs-C_3_N_4_−0.1 composites exhibit much better photocatalytic performance and more significant O_2_ promotion effect than corresponding TiO_2_ NCs. The produced liquid-phase oxygenates are CH_3_OH and HCOOH over TiO_2_{001}-C_3_N_4_−0.1, CH_3_OH and CH_3_OOH over TiO_2_{100}-C_3_N_4_−0.1, and CH_3_OOH over TiO_2_{101}-C_3_N_4_−0.1. Over TiO_2_{001}-C_3_N_4_−0.1 (Fig. 1g), the O_2_ addition increases the methane conversion rate from 358.5 to 696.3 μmol g_catalyst_^−1^ h^−1^, the selectivity of liquid-phase oxygenates and HCOOH respectively from 93.7% to 97.0% and from 56.4% to 69.8%, the HCOOH yield from 202.2 to 486 μmol g_catalyst_^−1^ h^−1^, and the H_2_O_2_ utilization efficiency from 53.4% to 93.3%.

The above results demonstrate an interesting photocatalytic system for efficiently converting CH_4_ to liquid-phase oxygenates in the presence of H_2_O_2_ and O_2_ at room temperature and ambient pressure over oxide-based photocatalysts, which presents high H_2_O_2_ utilization efficiencies due to the suppress effect of O_2_ on photocatalytic H_2_O_2_ decomposition into O_2_. The best photocatalyst, TiO_2_{001}-C_3_N_4_−0.1, exhibits an unprecedented H_2_O_2_ utilization efficiency of 93.3%, leading to a liquid-phase oxygenates selectivity of 97% and formic selectivity and yield respectively of 69.8% and 486 μmol_HCOOH_·g_catalyst_^−1^ h^−1^. Its apparent quantum efficiency at 365 nm was measured to be 0.48%.

TiO_2_{001}-C_3_N_4_−0.1 is stable and its performance maintains well within six cycles of photocatalytic activity evaluations (Supplementary Fig. 4). Routine structural characterization results (Supplementary Fig. 5), including XPS, VB-XPS, UV-Vis spectra and photocurrent measurements, show few difference between the as-synthesized and used TiO_2_{001}-C_3_N_4_−0.1 catalysts.

### Reaction mechanism

The carbon balance was calculated above 96.7% for all studied photocatalytic reactions. Blank photocatalytic experiment of photocatalytic reaction in the presence of TiO_2_{001}-C_3_N_4_−0.1 but absence of CH_4_ in the reactant did not produce detectable C-contained products; meanwhile, using ^13^CH_4_, all C-contained products only contained ^13^C (Supplementary Fig. 6). Thus, all C-contained products exclusively form from CH_4_. Initial evolutions of reaction products as a function of reaction time were examined over TiO_2_{001}-C_3_N_4_−0.1 (Supplementary Table 11). At a reaction time of 10 min, CH_3_OOH, CH_3_OH and HCHO were detected, and CH_3_OOH was the major product. The CH_3_OOH, CH_3_OH and HCHO productions increased at a reaction time of 30 min, meanwhile, HCOOH and CH_3_CH_2_OH appeared. As a reaction time of 1 h, the CH_3_OOH production decreased and HCHO was not detected, whereas the CH_3_OH and HCOOH productions increased greatly and the CH_3_CH_2_OH production increased slightly, meanwhile, CH_3_COOH emerged. These observations suggest CH_3_OOH as the primary product and CH_3_OH, HCHO, HCOOH, CH_3_CH_2_OH and CH_3_COOH as the secondary products that are produced sequentially. Moreover, the reaction rate of HCHO seems to be faster than the formation rate.

**Fig. 2:**
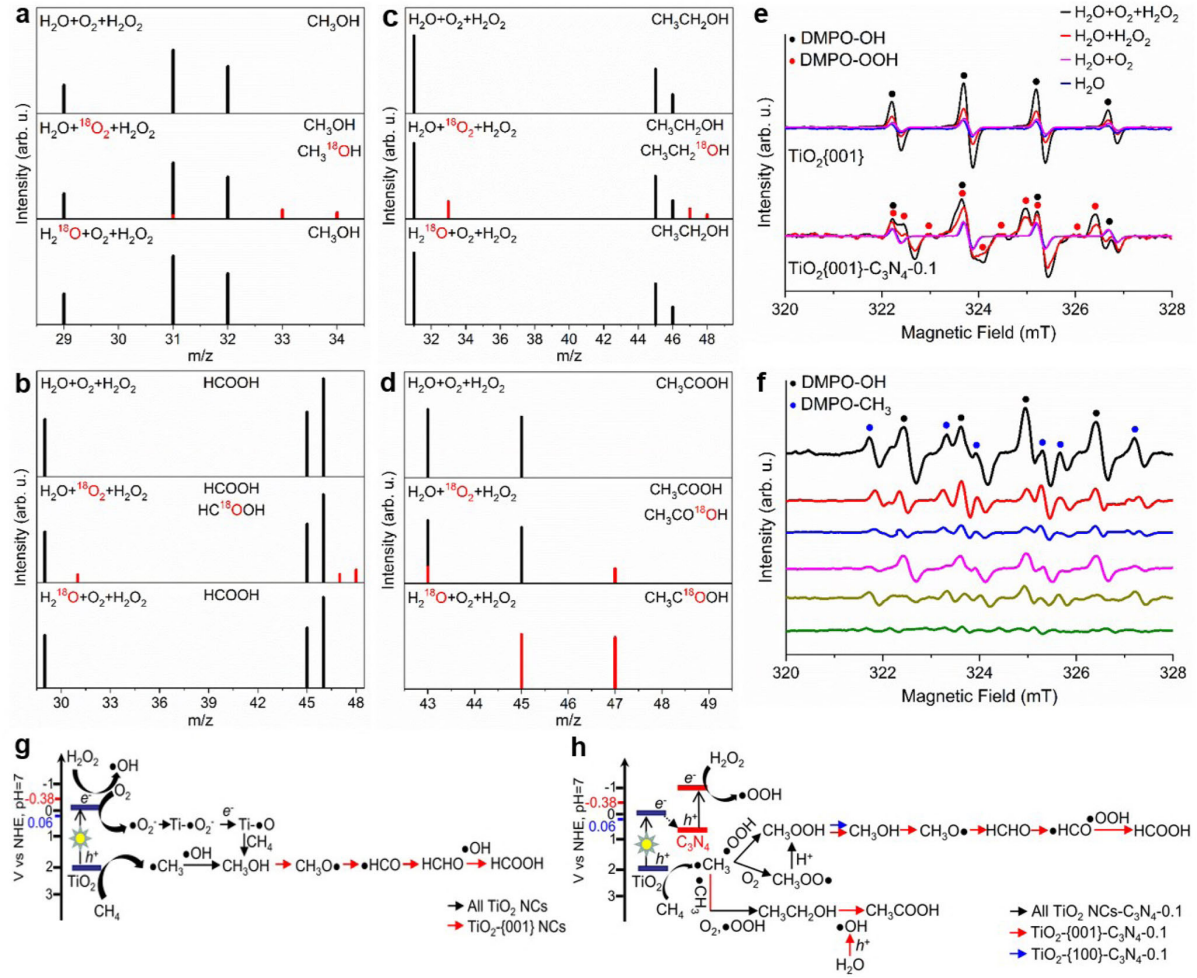
Reaction mechanism. Mass spectra of (**a**) methanol, (**b**) formic acid, (**c**) ethanol and (**d**) acetic acid formed during photocatalytic CH_4_ conversion over TiO_2_{001}-C_3_N_4_−0.1 under the reaction condition of 8%CH_4_ + 4%O_2_ + 88%Ar + 165 μL H_2_O_2_ + 20 mL H_2_O, 8%CH_4_ + 4%^18^O_2_ + 88%Ar + 165 μL H_2_O_2_ + 20 mL H_2_O, or 8%CH_4_ + 4%O_2_ + 88%Ar + 165 μL H_2_O_2_ + 20 mL H_2_^18^O. Photocatalyst amount: 20 mg; reaction temperature: 25 °C; reaction time: 8 h. **e** In situ ESR spectra of H_2_O, H_2_O + O_2_, H_2_O + H_2_O_2_ and H_2_O + O_2_ + H_2_O_2_ solutions under UV light illumination at 298 K in the presence of DMPO over TiO_2_{001} NCs and TiO_2_{001}-C_3_N_4_−0.1. **f** In situ ESR spectra of CH_4_ + H_2_O mixture under UV light illumination at 298 K in the presence of DMPO over TiO_2_{101} (olive), TiO_2_{100} (dark yellow) and TiO_2_{001} (magenta) NCs, TiO_2_{101}-C_3_N_4_−0.1 (blue), TiO_2_{100}-C_3_N_4_−0.1 (red) and TiO_2_{001}-C_3_N_4_−0.1 (black) composites. Schematic diagrams of proposed dominant photocatalytic aqueous-phase CH_4_ reaction paths to liquid-phase oxygenates in the presence of H_2_O_2_ and O_2_ over (**g**) TiO_2_ NCs and (**h**) TiO_2_ NCs-C_3_N_4_. The 0.06 eV and −0.38 eV refer to the redox potential of H_2_O_2_ activated to ·OH radicals and ·OOH radicals at pH = 7. Source data are provided as a Source Data file.

^18^O_2_ and H_2_^18^O were used to trace origins of oxygen atoms in the liquid-phase oxygenate products. ^18^O_2_ were observed to exert similar enhancement effects on the H_2_O_2_ utilization efficiency to ^16^O_2_ and to slightly affect the product selectivity (Supplementary Table 12). It is noteworthy that CH_3_OOH decomposes completely into CH_3_OH during the mass spectroscopy analysis (*13*). Over TiO_2_{001} NCs (Supplementary Figs. 7, 8), no ^18^O-labelled product was detected when H_2_^18^O was used, while CH_3_^18^OH and HC^18^O^16^OH were detected with CH_3_^18^OH/CH_3_^16^OH and HC^18^O^16^OH/HC^16^O^16^OH ratios respectively of around 0.12 and 0.11 when ^18^O_2_ was used. Over TiO_2_{001}-C_3_N_4_−0.1 (Fig. 2a–d and Supplementary Fig. 9), only CH_3_C^18^O^16^OH for CH_3_COOH and no other ^18^O-labelled oxygenate were detected when H_2_^18^O was used, while CH_3_^18^OH, HC^18^O^16^OH and CH_3_CH_2_^18^OH were detected with CH_3_^18^OH/CH_3_^16^OH, HC^18^O^16^OH/HC^16^O^16^OH and CH_3_CH_2_^18^OH/CH_3_CH_2_^16^OH ratios respectively of around 0.14, 0.13 and 0.25 when ^18^O_2_ was used, and CH_3_C^16^O^16^OH and CH_3_C^16^O^18^OH were detected for CH_3_COOH. Therefore, the oxygen atoms in CH_3_OOH, CH_3_OH, HCOOH and CH_3_CH_2_OH are contributed majorly by H_2_O_2_ and minor by O_2_, but seldom by H_2_O. Interestingly, HCOOH is formed via CH_3_OH oxidation exclusively by H_2_O_2_ whereas CH_3_COOH is formed via CH_3_CH_2_OH oxidation exclusively by H_2_O, suggesting that they follow different mechanisms. This was further supported by the observations that HC^16^O^16^OH/HC^18^O^16^OH and CH_3_C^18^O^16^OH/CH_3_C^18^O^18^OH were detected when ^18^O_2_ and H_2_^18^O were used (Supplementary Fig. 10). Photocatalytic CH_3_CH_2_OH oxidation with H_2_O to CH_3_COOH was reported to be mediated by ·OH radicals generated by photogenerated holes-participated activation of H_2_O, typically occurring in the aqueous solution^28,29^. Thus, photocatalytic reactions to other liquid-phase products occur on the photocatalyst surfaces. Meanwhile, only a tiny amount of C^16^O^18^O was detected in the gas phase products while no C^18^O and C^18^O_2_ was detected when ^18^O_2_ was used for both TiO_2_{001} NCs and TiO_2_{001}-C_3_N_4_−0.1 (Supplementary Fig. 11).

In order to further clarify the role of O_2_, the O_2_ concentration in the reactant was increased from 4% (8%CH_4_ + 4%O_2_ + 88%Ar + 165 μL H_2_O_2_ + 20 mL H_2_O) to 12% (8%CH_4_ + 12%O_2_ + 80%Ar+165 μL H_2_O_2_ + 20 mL H_2_O), and the photocatalytic reaction was studied over TiO_2_{001}-C_3_N_4_−0.1 comparatively with ^16^O_2_ or ^18^O_2_. Using ^16^O_2_ or ^18^O_2_ gave similar H_2_O_2_ utilization efficiencies of around 94% and slightly different CH_4_ conversion rates and product selectivity (Supplementary Table 13). Using ^18^O_2_, the CH_3_^18^OH/CH_3_^16^OH, HC^18^O^16^OH/HC^16^O^16^OH and CH_3_CH_2_^18^OH/CH_3_CH_2_^16^OH ratios in the liquid-phase products were measured respectively as around 0.19, 0.17 and 0.22 (Supplementary Figs. 12–14), similar to the case of the reactant with 4% O_2_; however, C^18^O and C^18^O_2_ were detected and the fraction of C^16^O^18^O in CO_2_ is much larger than that of C^16^O^16^O, different from the case of the reactant with 4% O_2_. Therefore, during photocatalytic aqueous-phase CH_4_ conversion in the presence of H_2_O_2_ and O_2_, CH_4_ preferentially reacts with H_2_O_2_ to produce liquid-phase oxygenates, while O_2_ acts mainly as a promoter to enhance H_2_O_2_ utilization efficiency and consequently CH_4_ conversion, and minorly as a reactant.

Using 5, 5-dimethyl-1-pyrroline N-oxide (DMPO) as the radical trapping agent, in situ EPR was used to probe radicals generated by photo-induced activation of various reactants. As shown in Fig. 2e and Supplementary Fig. 15, under UV light illumination, H_2_O is activated to ·OH radicals^30^ by photogenerated holes (*h*^*+*^) over various TiO_2_ NCs and TiO_2_ NCs-C_3_N_4_−0.1 composites, which barely changes upon the addition of O_2_. ·O_2_^−^ radicals formed by O_2_ activation with photogenerated electrons (*e*^−^) can not be observed in ESR spectra due to the instability in the aqueous solution, but their formation is evidenced by in situ ESR spectra in the methanol solution^31^ (Supplementary Fig. 16). Over TiO_2_ NCs, the ·OH radical signal grows slightly upon the addition of H_2_O_2_ and greatly upon the co-addition of H_2_O_2_ and O_2_. Over TiO_2_ NCs-C_3_N_4_−0.1 composites, the ·OH radical signal does not vary upon the addition of H_2_O_2_, while the ·OOH radical signal^25,26^ appears, and its intensity increases greatly upon the co-addition of H_2_O_2_ and O_2_. Thus, under UV light illumination, in addition to the *h*^*+*^-mediated decomposition into O_2_, H_2_O_2_ undergoes the *e*^−^-mediated activation into ·OH radicals over TiO_2_ NCs and ·OOH radicals over TiO_2_ NCs-C_3_N_4_−0.1 composites. The *e*^−^-mediated formation of dominant ·OOH radicals but few ·OH radicals over TiO_2_ NCs-C_3_N_4_−0.1 composites indicates that *e*^−^ for H_2_O_2_ activation is located on the conduction band mainly of C_3_N_4_ but seldom of TiO_2_, pointing to efficient interfacial transfer of *e*^−^ from the conduction band of TiO_2_ to the conduction band of C_3_N_4_. When CH_4_ was introduced to the aqueous solutions containing TiO_2_ NCs or TiO_2_ NCs-C_3_N_4_−0.1 composites under UV light illumination (Fig. 2f), ·CH_3_ radicals^22,26^, in addition to ·OH radicals, were detected. They greatly grew when isopropanol was added to quench ·OH radicals (Supplementary Fig. 17), but could not be detected in the presence of H_2_O_2_ and O_2_ when *h*^*+*^ was quenched using methanol (Supplementary Fig. 18). Thus, photocatalytic CH_4_ activation to ·CH_3_ radicals is mainly mediated by *h*^*+*^, instead of by·OOH, ·OH and ·O_2_^−^ radicals.

Based on the above isotope-labelled and ESR results, photocatalytic CH_4_ conversion with H_2_O_2_ is initiated by the reaction of *h*^*+*^-generated ·CH_3_ with *e*^−^-generated ·OH to CH_3_OH over TiO_2_ NCs (Fig. 2g) and with *e*^−^-generated ·OOH to CH_3_OOH over TiO_2_ NCs-C_3_N_4_−0.1 composites (Fig. 2h). The addition of O_2_ opens up minor reaction pathways, including the reaction of *h*^*+*^-generated ·CH_3_ with O_2_ to CH_3_OO· radicals that facilely transform to CH_3_OOH^22,32^ and the reaction of CH_4_ with Ti–O· formed by *e*^−^-mediated ·O_2_^−^ reactions on TiO_2_ surfaces directly to CH_3_OH^33^. We consider that the ·CH_3_ + O_2_ reaction occurs mainly for TiO_2_ NCs-C_3_N_4_−0.1 composites due to the lack of enough *e*^−^ on the TiO_2_ components while the CH_4_ + Ti–O· reaction occurs mainly for TiO_2_ NCs due to the absence of CH_3_OOH in the products. Moreover, the addition of O_2_ greatly enhances the intensities of ·OOH radicals over TiO_2_ NCs-C_3_N_4_−0.1 composites and ·OH radicals over TiO_2_ NCs formed by the *e*^−^-mediated H_2_O_2_ activation, and consequently the photocatalytic CH_4_ conversions. Since the presence of O_2_ efficiently suppresses the *h*^*+*^-mediated H_2_O_2_ decomposition to O_2_ under UV light illumination, the enhancement effect of O_2_ on ·OH and ·OOH generations from photocatalytic H_2_O_2_ activation is probably due to O_2_-suppressed *h*^*+*^-mediated H_2_O_2_ decomposition to O_2_ rather than O_2_-promoted *e*^−^-mediated H_2_O_2_ decomposition to ·OH and ·OOH radicals. O_2_ does not compete with H_2_O_2_ for *h*^*+*^ that is localized on the TiO_2_ surface, thus O_2_ likely suppresses H_2_O_2_ adsorption on TiO_2_, instead of reaction of adsorbed H_2_O_2_ with *h*^*+*^, to suppress the *h*^*+*^-mediated H_2_O_2_ decomposition to O_2_. Both TiO_2_ NCs and TiO_2_ NCs-C_3_N_4_−0.1 composites exhibit TiO_2_ facet-dependent intensities of various radicals. The ·OH radicals are strongest over TiO_2_{001} NCs among all TiO_2_ NCs and the ·OOH radicals are strongest over TiO_2_{001}-C_3_N_4_−0.1 composite among all TiO_2_ NCs-C_3_N_4_ composites (Supplementary Fig. 19). The ·CH_3_ radicals are strongest over TiO_2_{100} NCs among various TiO_2_ NCs and over TiO_2_{001}-C_3_N_4_−0.1 composite among various TiO_2_ NCs-C_3_N_4_ composites (Supplementary Fig. 20). Meanwhile, TiO_2_ NCs-C_3_N_4_ composites exhibit more reactive radicals than corresponding TiO_2_ NCs. These results are consistent with the results of photocatalytic activity.

The band structures of various TiO_2_ NCs and TiO_2_ NCs-C_3_N_4_−0.1 photocatalysts were determined using UV–vis spectra and valence band XPS spectra (Supplementary Fig. 21). TiO_2_ NCs-C_3_N_4_−0.1 exhibits smaller band gaps than corresponding the TiO_2_ NCs, suggesting stronger capacity for light absorption and charge generation. The conduction band edges of TiO_2_ NCs and TiO_2_ NCs-C_3_N_4_ composites were measured to be −0.14∼−0.34 and −0.41∼−0.47 vs *RHE*, respectively, consistent with the experimental observations that H_2_O_2_ undergoes the *e*^−^-mediated activation into ·OH radicals over TiO_2_ NCs and ·OOH radicals over TiO_2_ NCs-C_3_N_4_−0.1 composites (Fig. 2g, h). ESR spectra (Supplementary Fig. 22a) show that TiO_2_ NCs-C_3_N_4_−0.1 exhibit much lower densities of F^+^ and Ti^3+^ defects than TiO_2_ NCs and the defect density follows an order of TiO_2_{101} > TiO_2_{100} > TiO_2_{001} > TiO_2_{101}-C_3_N_4_−0.1 > TiO_2_{100}-C_3_N_4_−0.1 > TiO_2_{001}-C_3_N_4_−0.1. Accordingly, PL spectra (Supplementary Fig. 22b) show that the PL peak arising from the recombination of photoexcited electrons and holes displays an intensity order of TiO_2_{101} > TiO_2_{100} > TiO_2_{001} > TiO_2_{101}-C_3_N_4_−0.1 > TiO_2_{100}-C_3_N_4_−0.1 > TiO_2_{001}-C_3_N_4_−0.1. EIS spectra of various TiO_2_ NCs and TiO_2_ NCs-C_3_N_4_−0.1 photocatalysts were also measured, in which a smaller radius represents a low charge transfer resistance. All photocatalysts exhibit semicircle EIS spectra (Supplementary Fig. 22c), and the semicircle radius and consequently the charge transfer resistance follow an order of TiO_2_{101} > TiO_2_{100} > TiO_2_{001} > TiO_2_{101}-C_3_N_4_−0.1 > TiO_2_{100}-C_3_N_4_−0.1 > TiO_2_{001}-C_3_N_4_−0.1. ESR, PL and EIS are all bulk-sensitive characterization techniques, and their characterization results show that TiO_2_ NCs-C_3_N_4_−0.1 exhibit higher charge separation and transfer efficiencies than corresponding TiO_2_ NCs and that TiO_2_{001} is the best of various TiO_2_ NCs while TiO_2_{001}-C_3_N_4_−0.1 is the best of TiO_2_ NCs-C_3_N_4_−0.1 composite photocatalysts, consistent with the photocatalytic activity results.

**Fig. 3:**
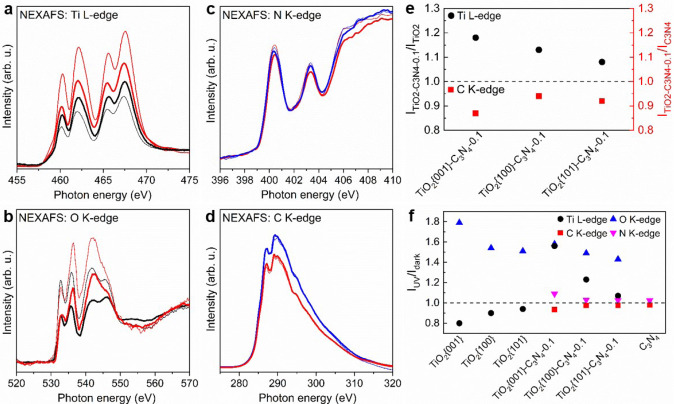
Interfacial charge transfer. **a** Ti L-edge, (**b**) O K-edge, (**c**) N K-edge and (**d**) C K-edge NEXAFS spectra of TiO_2_{001} NCs (black line), TiO_2_{001} NCs-C_3_N_4_−0.1 composite (red line) and C_3_N_4_ (blue line) in dark (thick line) and under UV light illumination (thin line). **e** Ti L-edge intensity ratios of TiO_2_ NCs-C_3_N_4_−0.1 composites against corresponding TiO_2_ NCs and C K-edge intensity ratios of TiO_2_ NCs-C_3_N_4_−0.1 composites against C_3_N_4_. **f** Intensity ratios of Ti L-edge, O K-edge, C K-edge and N K-edge features of various photocatalysts under UV light illumination against in dark. Source data are provided as a Source Data file.

NEXAFS acquired in a mode of total electron yield is a surface sensitive technique to probe the density of states of the orbitals involved in the electron transitions. UV light illumination excites electrons from the valence band to the conduction band, which consequently changes the density of states of the involved orbitals. We thus measured Ti L-edge, O K-edge, N K-edge and C K-edge NEXAFS spectra under dark and UV light illumination conditions of various samples (Fig. 3a–d, Supplementary Figs. 23, 24). The valence and conduction bands of TiO_2_ consist of the O 2*p* and Ti 3*d* orbitals, respectively, and the Ti L-edge and O K-edge NEXAFS features arise from the Ti 2*p*→3*d* and O 1 *s*→2*p* electron transitions, respectively. The valence and conduction bands of C_3_N_4_ consist of the N 2*p* and C 2*p* orbitals, respectively, and the N K-edge and C K-edge NEXAFS features arise from the N 1 *s*→2*p* and C 1 *s*→2*p* electron transitions, respectively. TiO_2_ NCs-C_3_N_4_ composites exhibit enhanced Ti L-edge and O K-edge features than corresponding TiO_2_ NCs but weakened C K-edge and N K-edge NEXAFS features than C_3_N_4_. This indicates an occurrence of TiO_2_→C_3_N_4_ electron transfer within TiO_2_ NCs-C_3_N_4_ composites, which decreases the electron density on TiO_2_ but increases the electron density of C_3_N_4_. Using the Ti L-edge and C K-edge NEXAFS features as examples (Fig. 3e), TiO_2_{001}-C_3_N_4_ composite exhibits the largest intensity variations of both Ti-L edge and C-K edge absorption features among all TiO_2_ NCs-C_3_N_4_ composites, demonstrating the most extensive electron transfer from TiO_2_{001} NCs to C_3_N_4_.

UV light illumination excites electrons from the valence bands of TiO_2_ or C_3_N_4_ to the conduction bands, and consequently results in weakened Ti L-edge NEXAFS features of TiO_2_ NCs, enhanced O K-edge NEXAFS features of TiO_2_ NCs and TiO_2_ NCs-C_3_N_4_−0.1 composites, and weakened C K-edge and enhanced N K-edge NEXAFS features of C_3_N_4_ and TiO_2_ NCs-C_3_N_4_−0.1 composites. But TiO_2_ NCs-C_3_N_4_−0.1 composites exhibit stronger Ti L-edge NEXAFS features under UV light illumination than under dark condition. This supports the formations of Z-scheme TiO_2_-C_3_N_4_ heterojunctions within TiO_2_ NCs-C_3_N_4_−0.1 composites^34^, in which the photogenerated electrons on the conduction band of TiO_2_ (Ti 3*d* orbital) efficiently transfer to the valence band of C_3_N_4_ (N 2*p* orbital) and recombine with photogenerated holes therein (Fig. 2h). Moreover, the total transferred electrons from the conduction band of TiO_2_ are more than the photogenerated electrons, likely due to a large number of photogenerated holes in the valence band of C_3_N_4_, which results in a less-occupied Ti 3*d* orbital and consequently a stronger Ti L-edge NEXAFS features of TiO_2_-C_3_N_4_−0.1 composites under UV light illumination than under dark condition. Figure 3f presents the ratios (I_UV_/I_dark_) of Ti L-edge, O K-edge, N K-edge and C K-edge NEXAFS features of various photocatalysts under UV light illumination against in dark, whose deviations from the unity reflect the photogenerated charges on the photocatalyst surfaces. Much larger concentrations of photogenerated electrons and holes are present on TiO_2_ surfaces than on C_3_N_4_ surface, suggesting more efficient charge separation and migration to surface within TiO_2_ NCs. C_3_N_4_ surface exhibits similar concentrations of photogenerated electrons and holes while TiO_2_ surfaces exhibit larger concentrations of photogenerated holes than of photogenerated electrons. TiO_2_ NCs-C_3_N_4_−0.1 composite surfaces exhibit slightly smaller concentrations of photogenerated holes than corresponding TiO_2_ NCs surfaces but larger concentrations of photogenerated charges than C_3_N_4_ surfaces. Thus, the Z-scheme TiO_2_-C_3_N_4_ heterojunctions within TiO_2_ NCs-C_3_N_4_−0.1 composites contribute to the charge separation and migration to surface over C_3_N_4_ component more than over TiO_2_ component. Among various TiO_2_ NCs or TiO_2_ NCs-C_3_N_4_−0.1 composites, the photocatalysts consisting TiO_2_{001} NCs exhibit the largest concentrations of photogenerated charges on the surfaces, leading to the largest concentrations of ·OH radicals over TiO_2_{001} NCs, ·OOH and ·CH_3_ radicals over TiO_2_{001}-C_3_N_4_ composite. However, TiO_2_{100} NCs, instead of TiO_2_{001} NCs, exhibit the largest concentration of ·CH_3_ radicals, which is likely relevant to the adsorption behaviors of CH_4_ on various photocatalysts. The adsorption heats of CH_4_ were measured similar for various TiO_2_ NCs (16.8–17.7 kJ/mol) or TiO_2_ NCs-C_3_N_4_−0.1 composites (11.1–14.5 kJ/mol) (Supplementary Figs. 25–27), while the adsorption amounts followed orders of TiO_2_{100} > TiO_2_{101} > TiO_2_{001} and of TiO_2_{001}-C_3_N_4_ > TiO_2_{100}-C_3_N_4_ > TiO_2_{101}-C_3_N_4_.

**Fig. 4:**
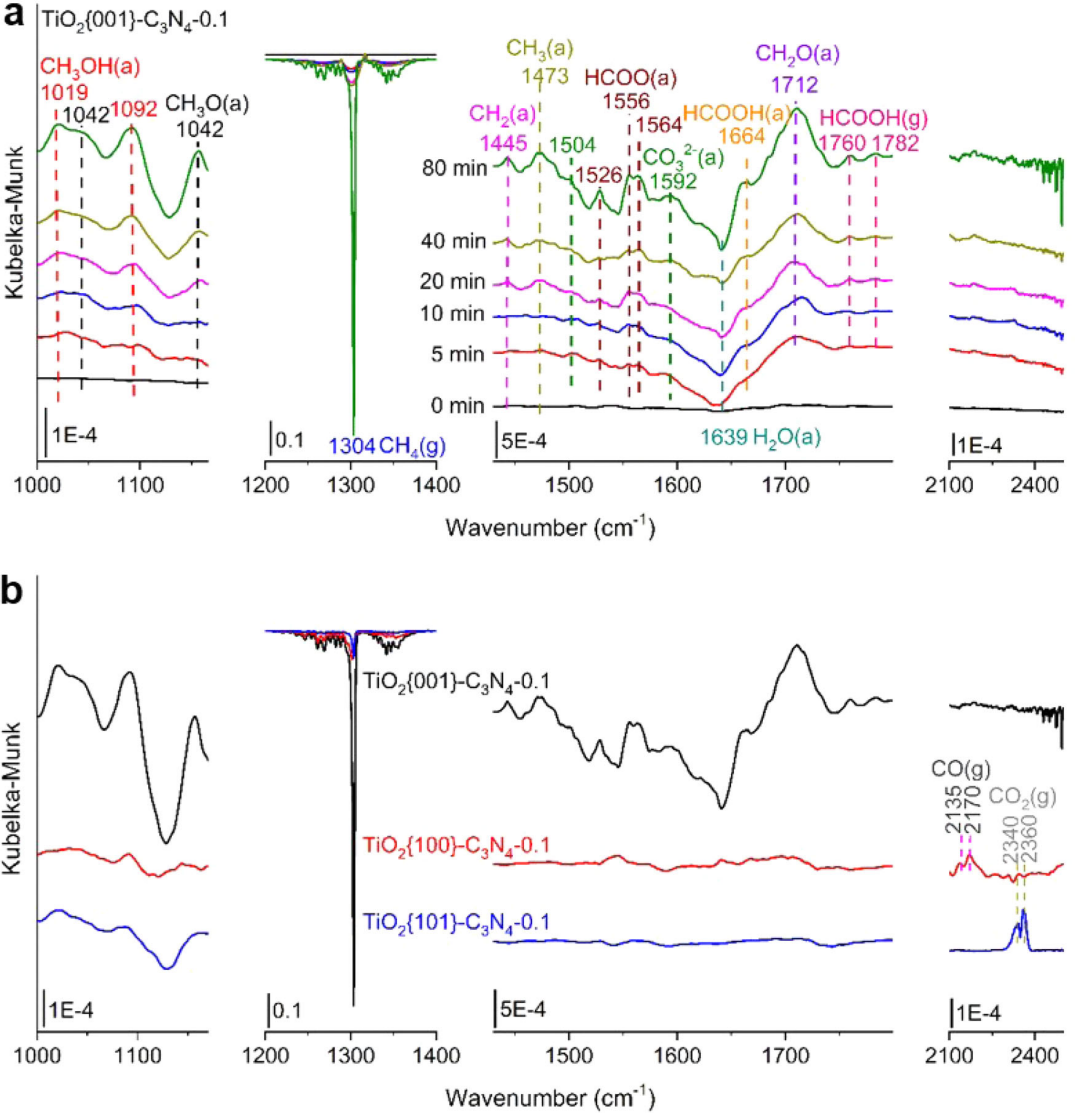
In situ characterization. **a** In situ DRIFTS spectra of photocatalytic CH_4_ conversion at 298 K under different light irradiation times over TiO_2_{001}-C_3_N_4_−0.1 with DRIFTS spectra prior to UV light illumination as the background spectra. **b** In situ DRIFTS spectra of photocatalytic CH_4_ conversion at 298 K under light irradiation for 80 min over TiO_2_{001}-C_3_N_4_−0.1, TiO_2_{100}-C_3_N_4_−0.1 and TiO_2_{101}-C_3_N_4_−0.1 with DRIFTS spectra prior to UV light illumination as the background spectra. Source data are provided as a Source Data file.

Various TiO_2_ NCs and TiO_2_ NCs-C_3_N_4_−0.1 composites show not only TiO_2_ facet-dependent activity but also TiO_2_ facet-dependent selectivity in photocatalytic CH_4_ conversion with H_2_O_2_ or H_2_O_2_ + O_2_. The photocatalysts with low photocatalytic activity exhibit low selectivity toward the liquid-phase products because more oxidizing radicals are available to eventually convert the liquid-phase intermediates to CO_2_. TiO_2_{001} NCs and TiO_2_{001}-C_3_N_4_−0.1 composite exhibit the highest photocatalytic activity and consequently the highest photocatalytic selectivity toward the liquid-phase products among various TiO_2_ NCs and various TiO_2_ NCs-C_3_N_4_−0.1 composites, respectively. Moreover, distributions of liquid-phase products vary with the TiO_2_ facets. Especially, HCOOH is the major liquid-phase product for the photocatalysts containing TiO_2_{001} NCs, but is barely observed for the photocatalysts containing TiO_2_{101} or TiO_2_{100} NCs. In situ DRIFTS spectra were used to explore surface reaction mechanisms of photocatalytic CH_4_ conversion with H_2_O_2_ + O_2_ over TiO_2_ NCs-C_3_N_4_−0.1 composites (Fig. 4). The observed vibrational bands (Supplementary Table 14) were assigned based on in situ DRIFTS spectra of CH_3_OH and HCOOH adsorption on various TiO_2_ NCs (Supplementary Fig. 28) and previous reports^35–39^. As the photocatalytic reaction prolongs over TiO_2_{001}-C_3_N_4_−0.1 composite (Fig. 4a), the vibrational features of adsorbed CH_3_ (1473 cm^−1^), CH_2_ (1445 cm^−1^), CH_3_OH (1019 and 1092 cm^−1^), CH_3_O (1042 and 1156 cm^−1^), CH_2_O (1712 cm^−1^), HCOO (1526, 1556 and 1564 cm^−1^), HCOOH (1664 cm^−1^) and carbonates (1504 and 1592 cm^−1^) species and gaseous HCOOH (1760 and 1782 cm^−1^) emerge and grow at the expense of gaseous CH_4_ (1304 cm^−1^). These results directly evidence the occurrences of photocatalytic oxidation of CH_4_ to CH_3_OH via the CH_3_ intermediate and further to HCOOH via the CH_3_O, CH_2_O and HCO (in the form of HCOO_TiO2_) intermediates, as schematically shown in Fig. 2g, h. Although the carbonate intermediates were observed, no signals of CO or CO_2_ appeared, indicating that the carbonate intermediates are very stable on TiO_2_{001}-C_3_N_4_ composite. Comparing TiO_2_{001}-C_3_N_4_−0.1 composite, TiO_2_{100}-C_3_N_4_−0.1 and TiO_2_{101}-C_3_N_4_−0.1 composites exhibit very different in situ DRIFTS spectra (Fig. 4b). The gaseous CH_4_ consumptions and the CH_3_OH(a) formation are greatly smaller over TiO_2_{100}-C_3_N_4_−0.1 and TiO_2_{101}-C_3_N_4_−0.1 composites than over TiO_2_{001}-C_3_N_4_−0.1 composite. Meanwhile, only very minor vibrational features of surface intermediates appear whereas obvious vibrational features of gaseous CO (2135 and 2170 cm^−1^) and CO_2_ (2340 and 2360 cm^−1^) emerge over TiO_2_{100}-C_3_N_4_−0.1 and TiO_2_{101}-C_3_N_4_−0.1 composites, respectively. These in situ DRIFTS results are consistent with the photocatalytic reaction data that TiO_2_{001}-C_3_N_4_−0.1 composite are much more photocatalytic active and selective toward the liquid-phase products in photocatalytic CH_4_ conversion with H_2_O_2_ + O_2_ than TiO_2_{100}-C_3_N_4_−0.1 and TiO_2_{101}-C_3_N_4_−0.1 composites.

### Theoretical calculations

**Fig. 5:**
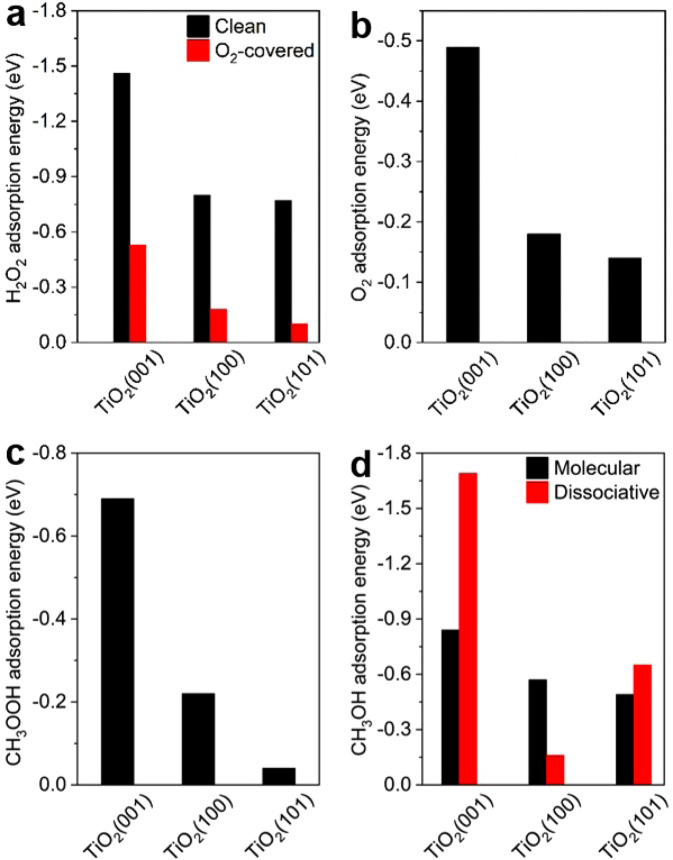
DFT calculations. Calculated adsorption energies of (**a**) H_2_O_2_ on clean and O_2_-covered TiO_2_ (001), (100) and (101) surfaces, (**b**) O_2_, (**c**) CH_3_COOH and (**d**) molecular and dissociative CH_3_OH adsorption on TiO_2_ (001), (100) and (101) surfaces. Source data are provided as a Source Data file.

DFT calculations were carried out to understand O_2_-suppressed photocatalytic H_2_O_2_ decomposition to O_2_ and facet-dependent photocatalytic selectivity of CH_4_. Since both photocatalytic H_2_O_2_ decomposition to O_2_ and photocatalytic CH_4_ conversion are mediated by photogenerated holes located predominantly on TiO_2_ NCs, thus we considered TiO_2_ facets, but not TiO_2_-C_3_N_4_ interfaces, during the DFT calculations. As reported previously^35,40–44^, the anatase TiO_2_(001) surface exposed on TiO_2_{001} NCs exhibits a typical reconstructed (001)-(1 × 4) surface with fourfold-coordinated Ti cations (Ti_4c_) at the (1 × 4) added row, fivefold-coordinated Ti cations (Ti_5c_) at the (1 × 1) basal surface and twofold-coordinated O anions (O_2c_), the anatase TiO_2_(100) surface exposed on TiO_2_{100} NCs exhibits a typical reconstructed (1 × 2) surface with the Ti_5c_, O_2c_ and threefold-coordinated O (O_3c_) sites, and the anatase TiO_2_(101) surface exposed on TiO_2_{101} NCs exhibits a (1 × 1) unreconstructed surface with the Ti_5c_, O_2c_ and O_3c_ sites (Supplementary Fig. 29). The Ti_4c_ sites on TiO_2_(001) surface show much stronger adsorption ability than the Ti_5c_ sites on TiO_2_ (001), (100) and (101) surfaces. As shown in Fig. 5a and Supplementary Fig. 30, the adsorption energy of H_2_O_2_ is −1.46, −0.80 and −0.77 eV on TiO_2_ (001), (100) and (101) surfaces, respectively, and greatly decreases to −0.53, −0.18 and −0.10 eV on O_2_-covered TiO_2_ (001), (100) and (101) surfaces, respectively. The adsorption energy of O_2_ is −0.49, −0.18 and −0.14 eV on TiO_2_ (001), (100) and (101) surfaces, respectively (Fig. 5b and Supplementary Fig. 31). These DFT calculation results demonstrate that O_2_ is capable of weakening H_2_O_2_ adsorption on TiO_2_ to suppress the *h*^*+*^-mediated H_2_O_2_ decomposition to O_2_. The strongest adsorption of O_2_ on TiO_2_(001) surface exerts the strongest suppress effect on H_2_O_2_ decomposition to O_2_ on TiO_2_{001} NCs. CH_4_ adsorption on TiO_2_ (001), (100) and (101) surfaces are very weak with an adsorption energy of −0.17, −0.03 and −0.04 eV (Supplementary Fig. 32). Adsorption energy of CH_3_OOH on TiO_2_ (001), (100) and (101) surfaces is −0.69, −0.22 and −0.04 eV, respectively (Fig. 5c and Supplementary Fig. 33). CH_3_OH adsorbs both molecularly and dissociatively with adsorption energy respectively of −0.84 and −1.69 eV on TiO_2_ (001) surface, −0.57 and −0.16 eV on TiO_2_ (100) surface, −0.49 and −0.65 eV on TiO_2_(101) surface (Fig. 5d and Supplementary Fig. 34). The calculated adsorption energies of various liquid-phase products on TiO_2_ (001), (100) and (101) surfaces are consistent with the experimentally observed different selectivity toward liquid-phase products in photocatalytic CH_4_ conversion over TiO_2_ {001}, {100} and {101} NCs, suggesting that desorption of various products from TiO_2_ surface play a key role in determining the selectivity. Preferential dissociation of produced CH_3_OH on TiO_2_{001} NCs and TiO_2_{001}-C_3_N_4_−0.1 composite forms methoxy species which is further photooxidized to HCOOH (Fig. 2g, h), leading to the experimental results that HCOOH is the major liquid-phase product. The very weak adsorption of produced CH_3_OOH on TiO_2_(101) surface makes it as the sole liquid-phase product over TiO_2_{101}-C_3_N_4_−0.1 composite.

## Discussion

Therefore, O_2_ is a general and efficient molecular additive to suppress H_2_O_2_ adsorption on oxide photocatalysts and consequently photogenerated holes-mediated H_2_O_2_ decomposition to O_2_ during photocatalytic reactions. Such a suppress effect, together with efficient charge separation within TiO_2_{001}-C_3_N_4_ heterojunctions, photogenerated holes-mediated activation of CH_4_ into ·CH_3_ radicals on TiO_2_{001} and photogenerated electrons-mediated activation of H_2_O_2_ into ·OOH radicals on C_3_N_4_, and preferential dissociative adsorption of methanol on TiO_2_{001}, leads to an unprecedented high H_2_O_2_ utilization efficiency of 93.3% and highly active and selective to liquid-phase oxygenates with formic acid as the major product during photocatalytic conversion of methane with H_2_O_2_ and O_2_. H_2_O_2_ production is known as an environment-unfriendly and economic-costly process^45^, therefore, our findings point to co-use of H_2_O_2_ and O_2_ in photocatalytic oxidation reactions over oxide-based photocatalysts as a promising strategy to achieve high H_2_O_2_ utilization efficiency and excellent photocatalytic performance.

## Methods

### Materials

H_2_O_2_ aqueous solution (20 wt.%), HF aqueous solution (40 wt.%), acetate, acetic acid, methanol, isopropanol, Ti(OBu)_4_, K_2_TiO(C_2_O_4_)_2_, P25, ZnO, Fe_2_O_3_, WO_3_ and V_2_O_5_ were all with the analytical grade and purchased from Sinopharm Chemical Reagent Co. CuO (≥99%), dicyandiamide (≥98%), pentane-2,4-dione (≥98%), 5, 5-dimethyl-1-pyrroline N-oxide (DMPO) (≥97%) and 3-(trimethylsilyl)−1-propanesulfonic acid sodium salt (DSS) (≥97%) were purchased from Sinopharm Chemical Reagent Co. Reactants of CH_4_ (8%) + O_2_(4%) + Ar (88%) and CH_4_ (8%) + O_2_ (4%) + O_2_ (10%) + Ar (78%) were purchased from Nanjing Shang Yuan Industry Factory. ^13^CH_4_ (^13^C enrichment > 99%atom), ^18^O_2_ (^18^O enrichment ≥ 98%atom) and H_2_^18^O (^18^O enrichment ≥ 98%atom) were purchased from Wuhan Newradar Gas Co. All chemicals and gases were used as received.

### Catalyst synthesis

TiO_2_ NCs predominantly exposing different types of facets were prepared following previous procedures^27^.

Synthesis of anatase TiO_2_{001} NCs: typically, 25 mL Ti(OBu)_4_ and 3 mL HF aqueous solution (40 wt%) were mixed under stirring at RT (Caution: Hydrofluoric acid (HF) is extremely corrosive and a contact poison, and it should be handled with extreme care! Hydrofluoric acid solution is stored in Teflon containers in use.). The solution was then transferred into a 50 mL Teflon lined stainless steel autoclave and kept at 180 °C for 24 h. The resulted white precipitate was collected by centrifugation, washed repeatedly with ethanol and water, and dried at 70 °C for 12 h. The acquired powder was dispersed in 700 mL NaOH aqueous solution (0.1 mol/L), stirred for 24 h at RT, centrifuged, and washed repeatedly with water until the pH value of aqueous solution was of 7–8.

Synthesis of anatase TiO_2_{100} and TiO_2_{101} NCs: typically, 6.6 mL TiCl_4_ was added dropwise into 20 mL HCl aqueous solution (0.43 mol/L) at 0 °C. After stirring for an additional 0.5 h, the solution was added dropwise into 50 mL NH_3_ aqueous solution (5.5 wt%) under stirring at RT. Then the pH value of the solution was adjusted to between 6 and 7 using 4 wt% NH_3_ aqueous solution, after which the system was stirred at RT for 2 h. The resulted precipitate was filtered, washed repeatedly with water until no residual Cl^−^ could be detected, and then dried at 70 °C for 12 h to acquire Ti(OH)_4_ precursor. To prepare anatase TiO_2_-{100} nanocrystals, 2.0 g Ti(OH)_4_ and 0.5 g (NH_4_)_2_SO_4_ were dispersed in a mixture of 15 mL H_2_O and 15 mL isopropanol under stirring at RT, then the mixture was transferred into a 50 mL Teflon-lined stainless steel autoclave and kept at 180 °C for 24 h. The obtained white precipitate was collected and washed repeatedly with water. To prepare anatase TiO_2_-{101} nanocrystals, 2.0 g Ti(OH)_4_ and 0.2 g NH_4_Cl were dispersed in a mixture of 15 mL H_2_O and 15 mL isopropanol under stirring at RT, then the mixture was transferred into a 50 mL Teflon-lined stainless steel autoclave and kept at 180 °C for 24 h. The obtained white precipitate was collected and washed repeatedly with water.

Synthesis of anatase TiO_2_ NCs-C_3_N_4_ composites: calculated amounts of dicyandiamide (C_2_H_4_N_4_) and TiO_2_ NCs were mixed in a crucible. The crucible was placed into a tube furnace, purged in Ar 1 h, and heated to 550 °C at a rate of 2.5 °C/min and kept for 4 h, then cooled to room temperature. The acquired powders were taken out and grind to obtain TiO_2_ NCs-C_3_N_4_ composites.

### Structure characterizations

Powder X-ray diffraction (XRD) patterns were recorded on a Philips X’Pert Pro Super diffractometer with Cu Kα radiation (λ = 0.15406 nm) operated at 40 kV and 50 mA. Transmission infrared spectra were recorded on a Nicolet 8700 spectrometer at room temperature. Electron paramagnetic resonance (ESR) spectra with and without Xenon lamp irradiation were recorded on a JEOL JES-FA200 ESR spectrometer (9.063 GHz, X-band) at 130 K with employed microwave power, modulation frequency, and modulation amplitude of 0.998 mW, 100 kHz, and 0.35 mT, respectively. Steady-state photoluminescence spectra were measured on a HORIBA LabRAM HR spectrograph with a continuous wave 325 nm laser as the exciting source and the signal was collected by passing through a filter with cut-off wavelengths below 380 nm. UV–vis diffuse reflectance spectra (UV–vis DRS) were obtained on a Shimadzu DUV-3700 spectrophotometer equipped with an integrating sphere attachment. X-ray photoelectron spectroscopy (XPS) measurements were performed on an ESCALAB 250 high-performance electron spectrometer using monochromatized Al Kα (hv = 1486.7 eV) as the excitation source, and the likely charging of samples was corrected by setting the C 1 *s* binding energy of the adventitious carbon to 284.8 eV. Near-edge X-ray absorption fine structure (NEXAFS) spectra were measured at photoelectron spectroscopy end-station of National Synchrotron Radiation Laboratory. Transmission electron microscopy (TEM), high-resolution transmission electron microscopy (HRTEM) and element mapping images were performed with a JEOL JEM-2100F instrument at an acceleration voltage of 120 kV.

Adsorption microcalorimetry measurements were carried out on a home-setup equipment consisting of a Setaram Sensys EVO 600 Tian-Calvet microcalorimeter and an Micromeritics Autochem II 2920 automated chemisorption apparatus^46^. Typically, 50 mg sample was placed in the sample quartz tube and degassed in He (flow rate: 50 mL/min) at 200 °C for 60 min, then the sample was cooled to −100 °C, and the gas stream was switched to 2% CH_4_/He (flow rate: 50 mL/min) for adsorption. After CH_4_ adsorption reached saturation, the gas stream was switched back to He (flow rate: 50 mL/min) for desorption. The adsorption/desorption amounts and accompanying heat flows were quantified by the chemisorption apparatus and microcalorimeter, respectively, from which the adsorption/desorption heats were calculated.

In situ DRIFTS experiments were performed at 298 K on a Thermo Scientific Nicolet iS50 FTIR Spectrometer with a mercury cadmium telluride detector cooled with liquid nitrogen. The spectrometer was equipped with a Harrik Praying Mantis diffuse reflection accessory and a Harrick high-temperature reaction cell with ZnSe windows. The reaction cell was connected to a SH-110 dry scroll vacuum pump (Agilent Technologies), H_2_O_2_ aqueous solution stored in a quartz tube welded with Kovar, and 8%CH_4_ + 4%O_2_ + 88%Ar gas via three closed valves. The 30% H_2_O_2_ aqueous solution was purified by repeated cycles of freeze−pump−thaw treatments. The UV light irradiation on the sample was accomplished through the front window of the high-temperature reaction chamber using a 100 W high-pressure Hg arc lamp (Oriel 6281), which provides a pressure-broadened emission spectrum from gaseous Hg in the UV-light region. A water filter was used to remove the IR portion of the emission spectrum. Typically, the sample was loaded in the sample holder of the reaction cell, then the reaction cell was evacuated by opening the valve connecting the vacuum pump. After the pressure decreased to 10 Torr, the valve connecting the vacuum pump was closed, and the valve connecting the H_2_O_2_ aqueous solution was opened to reach a stable pressure, and then the valve connecting to 8%CH_4_ + 4%O_2_ + 88%Ar gas was open to allow the pressure of the reaction cell to 1 atm, and finally both valves were closed. The DRIFTS spectrum of the sample prior to UV light illumination was firstly taken as the background spectrum, then the UV light was turned on to irradiate the sample and the DRIFTS spectra were taken in a sequential mode. The DRIFTS spectrum of the sample was also taken after the turn off of the UV light. All DRIFTS spectra were measured with 128 scans at a resolution of 4 cm^−1^.

### Photocatalytic activity measurements

Photocatalytic activity of various samples in aqueous-phase methane conversion was evaluated in a quartz reactor with a cooling-water jacket to maintain the reaction temperature at 25 °C under atmospheric pressure using a 300 W Xe lamp as the light source whose spectrum is shown in the Supplementary Fig. 35. Typically, 20 mg photocatalyst, 20 mL deionized water and a certain amount of H_2_O_2_ aqueous (1 mol/L) solution were mixed in the reactor. The reaction system was adequately deaerated by reaction gas for 1 h, and then was irradiated by the Xe lamp. then the photocatalytic reaction was carried out. After a desirable reaction time, 0.5 mL gas was sampled from the reaction system and its composition products was analyzed by a Fuli GC9720 gas chromatography equipped with FID and TCD detectors.

Liquid-phase oxygenate products were analyzed and quantified by ^1^H nuclear magnetic resonance (NMR) spectra acquired on a JEOL ECS 400 MHz NMR spectrometer. A DSS solution in D_2_O (0.020 wt.%) with the ^1^H chemical shift at δ = 0.0 ppm was prepared to calibrate the chemical shift. Typically, 0.70 mL clear aqueous solution was sampled from the reaction system and mixed with 0.10 mL DSS solution in a NMR tube and the ^1^H NMR spectrum was taken. The intensity of measured ^1^H NMR peak of various products were compared to the corresponding ^1^H NMR working curve acquired using pure product of different concentrations (Supplementary Fig. 36). Since pure CH_3_OOH could not be purchased while both CH_3_OOH and CH_3_OH have the methyl group, the amount of CH_3_OOH in the liquid-phase products was quantified using the working curve of CH_3_OH^22^.

The concentration of HCHO was quantified by the colorimetric method^22^. Typically, 100 mL of the reagent aqueous solution was prepared by dissolving 15 g ammonium acetate, 0.3 mL acetic acid, and 0.2 mL pentane-2,4-dione in water. Then, 0.5 mL liquid product was mixed with 2.0 mL water and 0.5 mL reagent solution. The mixed solution was maintained at 35 °C and measured by UV − vis absorption spectrum until the absorption intensity at 412 nm did not further increase. The concentration of HCHO in the liquid product was determined by the standard curve (Supplementary Fig. 37).

The concentration of H_2_O_2_ in the aqueous solution was quantified by the colorimetric method^13,21^. Typically, a reagent aqueous solution was prepared by dissolving 0.636 g K_2_TiO(C_2_O_4_)_2_ and 20 μL concentrated H_2_SO_4_ (98%) in 100 mL deionized water. 0.2 mL aqueous solution was exacted from the reaction system and mixed with 4.0 mL reagent solution. Then the UV − vis absorption spectrum of the mixed solution was measured, and the intensity of the absorption peak at 398 nm arising from the complex formed by K_2_TiO(C_2_O_4_)_2_ and H_2_O_2_ was compared to the working curve acquired using pure H_2_O_2_ aqueous solution of different concentrations (Supplementary Fig. 38) to quantify the H_2_O_2_ concentration.

Methane conversion, product selectivity, H_2_O_2_ conversion and H_2_O_2_ utilization efficiency were calculated as the following:

$$	{{{{{\rm{Methane}}}}}}\; {{{{{\rm{conversion}}}}}} \, (\%)=({{{{{\rm{n}}}}}}\left({{{{{\rm{CH}}}}}}_{4}\right)_{{{{{{\rm{before}}}}}}\; {{{{{\rm{reaction}}}}}}}\\ 	 \quad - {{{{{\rm{n}}}}}}({{{{{\rm{CH}}}}}}_{4})_{{{{{{\rm{after}}}}}}\; {{{{{\rm{reaction}}}}}}})/{{{{{\rm{n}}}}}}({{{{{\rm{CH}}}}}}_{4})_{{{{{{\rm{before}}}}}}\; {{{{{\rm{reaction}}}}}}}\times 100\%$$

$${{{{{\rm{Product}}}}}}\; {{{{{\rm{selectivity}}}}}} \, (\%)={{{{{{\rm{n}}}}}}}_{{{{{{\rm{Product}}}}}}}/({{{{{{\rm{n}}}}}}({{{{{{\rm{CH}}}}}}}_{4})}_{{{{{{\rm{before}}}}}}\; {{{{{\rm{reaction}}}}}}}-{{{{{{\rm{n}}}}}}({{{{{{\rm{CH}}}}}}}_{4})}_{{{{{{\rm{after}}}}}}\; {{{{{\rm{reaction}}}}}}})\times 100\%$$

$$	{{{{{{\rm{H}}}}}}}_{2}{{{{{{\rm{O}}}}}}}_{2}{{{{{\rm{conversion}}}}}} \, (\%)=\left({{{{{{\rm{n}}}}}}({{{{{{\rm{H}}}}}}}_{2}{{{{{{\rm{O}}}}}}}_{2})}_{{{{{{\rm{before}}}}}}\; {{{{{\rm{reaction}}}}}}}\right .\\ 	 \quad \left .- {{{{{{\rm{n}}}}}}({{{{{{\rm{H}}}}}}}_{2}{{{{{{\rm{O}}}}}}}_{2})}_{{{{{{\rm{after}}}}}}\; {{{{{\rm{reaction}}}}}}}\right)/{{{{{{\rm{n}}}}}}({{{{{{\rm{H}}}}}}}_{2}{{{{{{\rm{O}}}}}}}_{2})}_{{{{{{\rm{before}}}}}}\; {{{{{\rm{reaction}}}}}}}\times 100\%$$

$$	{{{{{{\rm{H}}}}}}}_{2}{{{{{{\rm{O}}}}}}}_{2}{{{{{\rm{utilization}}}}}}\; {{{{{\rm{efficiency}}}}}} \, (\%)=\big({{{{{\rm{n}}}}}}({{{{{{\rm{H}}}}}}}_{2}{{{{{{\rm{O}}}}}}}_{2})_{{{{{{\rm{before}}}}}}\; {{{{{\rm{reaction}}}}}}}\\ 	 \quad - {{{{{\rm{n}}}}}}({{{{{{\rm{H}}}}}}}_{2}{{{{{{\rm{O}}}}}}}_{2})_{{{{{{\rm{after}}}}}}\; {{{{{\rm{reaction}}}}}}}-{{{{{\rm{n}}}}}}({{{{{{\rm{H}}}}}}}_{2}{{{{{{\rm{O}}}}}}}_{2})_{{{{{{\rm{decomposition}}}}}} \; {{{{{\rm{to}}}}}} \; {{{{{\rm{O}}}}}}2}\big )/{{{{{\rm{n}}}}}}({{{{{{\rm{H}}}}}}}_{2}{{{{{{\rm{O}}}}}}}_{2})_{{{{{{\rm{before}}}}}} \; {{{{{\rm{reaction}}}}}}}\times 100\%$$

$${{{{{{\rm{n}}}}}}({{{{{{\rm{H}}}}}}}_{2}{{{{{{\rm{O}}}}}}}_{2})}_{{{{{{\rm{decomposition}}}}}}\; {{{{{\rm{to}}}}}} \; {{{{{\rm{O}}}}}}2}={{{{{{\rm{n}}}}}}({{{{{{\rm{O}}}}}}}_{2})}_{{{{{{\rm{after}}}}}} \; {{{{{\rm{reaction}}}}}}} - {{{{{{\rm{n}}}}}}({{{{{{\rm{O}}}}}}}_{2})}_{{{{{{\rm{before}}}}}} \; {{{{{\rm{reaction}}}}}}} - {{{{{{\rm{n}}}}}}}_{{{{{{\rm{O}}}}}}2{{{{{\rm{reacted}}}}}}}$$

$$	{{{{{\rm{Carbon}}}}}}\; {{{{{\rm{balance}}}}}} \, (\%)={{{{{{\rm{n}}}}}}}_{{{{{{\rm{carbon}}}}}}\; {{{{{\rm{in}}}}}}\; {{{{{\rm{all}}}}}}\; {{{{{\rm{products}}}}}}}/\\ 	 \quad ({{{{{{\rm{n}}}}}}({{{{{{\rm{CH}}}}}}}_{4})}_{{{{{{\rm{before}}}}}}\; {{{{{\rm{reaction}}}}}}}-{{{{{{\rm{n}}}}}}({{{{{{\rm{CH}}}}}}}_{4})}_{{{{{{\rm{after}}}}}}\; {{{{{\rm{reaction}}}}}}})\times 100\%$$

Where n was the quantified amount of reactants or products, while n_O2 reacted_ was calculated from the amount of products and the ratio of the products formed by O_2_ based on the isotope-labelling results. For photocatalytic reactions using ^18^O_2_, n_18O2 reacted_ was calculated by (n(^18^O_2_)_before reaction_-n(^18^O_2_)_after reaction_), in which n(^18^O_2_) was quantified using GC-MS. The carbon balance was calculated not less than 96.7% for all studied photocatalytic reactions.

### Product analysis of photocatalytic reactions using isotope-labelled reactants

Liquid-phase oxygenates produced by aqueous-phase photocatalytic methane conversion using ^13^CH_4_ were analyzed by ^1^H NMR and ^13^C with decoupling NMR spectrometer as described above. Products of aqueous-phase photocatalytic methane conversion using ^18^O_2_ and or H_2_^18^O were analyzed by mass spectrometer as the following: 0.5 mL gas was sampled from the reaction system and its composition was analyzed on a Trace GC/ISQ MS; and 3 mL clear aqueous solution was sampled from the reaction system and transferred into a quartz tube welded with Kovar and then connected to a QIC20 mass spectrometer (Heiden Analytical Ltd.) and a Hicube 80 Eco pump station by two closed valves. The aqueous solution was purified by repeated cycles of freeze−pump−thaw treatments and its composition was analyzed by the QIC20 mass spectrometer.

### Theoretical calculations

All theoretical calculations were carried out using the Vienna ab initio simulation package (VASP)^47,48^, and the exchange-correlation term was described by the Perdew, Burke and Ernzerhof version within the generalized gradient approximation (PBE-GGA)^49^. The project-augmented wave (PAW)^50,51^ method was used to represent the core-valence electron interaction. The titanium 3 *s*, 3*p*, 3*d*, 4 *s*, and the carbon and oxygen 2 *s*, 2*p* electrons were treated as valence electrons and an energy cutoff of 400 eV for the basis-set expansion was used. The anatase TiO_2_(001)-(1 × 4), TiO_2_(101) and TiO_2_(100) surface was modeled as a periodic slab with six O-Ti-O trilayers of oxide. A vacuum between slabs >15 Å and corresponding 1 × 1 × 1 k-point mesh were used during the calculations. Adsorption was modeled on one side of the slab, and during structural optimizations, all of the atoms except those in the bottom TiO_2_ trilayer of the slab, were allowed to relax until atomic forces reached below 0.05 eV/Å. The adsorption energy (*E*_ads_) was expressed using the average adsorption energy calculated by *E*_ads_ = *E*_ad∕sub_ – (*E*_sub_ + *E*_ad_) in which *E*_ad∕sub_ is the total energy of the interacting system containing adsorbed molecules and TiO_2_ support in a surface cell, *E*_sub_ is the total energy of the anatase TiO_2_ slab and *E*_ad_ is the total energy of the molecule in gas phase.

Details on structural characterizations, activity evaluations, and DFT calculations can be found in the supplementary information.

## Supplementary information

Supplementary Information

Peer Review File

## Data Availability

The data supporting the findings of the study are available within the paper and its Supplementary Information. Source data are provided with this paper.

## Source data

Source Data
