# Supplementary material for: Molecular oxygen enhances H2O2 utilization for the photocatalytic conversion of methane to liquid-phase oxygenates
Source: Nat Commun. 2022 Nov 5;13:6677. doi: 10.1038/s41467-022-34563-4 (PMC9637122; doi:10.1038/s41467-022-34563-4)
Supplement: Supplementary file 1 — Supplementary Information [file 41467_2022_34563_MOESM1_ESM.pdf]

Supplementary information

Molecular oxygen enhances H_2O_2 utilization for the photocatalytic conversion of methane to liquid-phase oxygenates

Xiao Sun,[1#] Xuanye Chen,[1#] Cong Fu,[1] Qingbo Yu,[2] Xu-Sheng Zheng,[3] Fei Fang,[1] Yuanxu Liu,[4] Junfa Zhu,[3] Wenhua Zhang,[1] Weixin Huang[1,5]*

[1] Hefei National Research Center for Physical Sciences at the Microscale, *i*ChEM, Key Laboratory of Surface and Interface Chemistry and Energy Catalysis of Anhui Higher Education Institutes, School of Chemistry and Materials Science, University of Science and Technology of China, Hefei 230026, China.

[2] Department of Materials Science and Engineering, Anhui University of Science and Technology, Huainan 232001, China.

[3] National Synchrotron Radiation Laboratory, University of Science and Technology of China, Hefei, Anhui 230029, China.

[4] School of Pharmacy, Anhui University of Chinese Medicine, Anhui Academy of Chinese Medicine, Hefei, Anhui 230012, China.

[5] Dalian National Laboratory for Clean Energy, Chinese Academy of Sciences, Dalian 116023, China.

[#] Equal contribution

* Correspondence author: huangwx@ustc.edu.cn

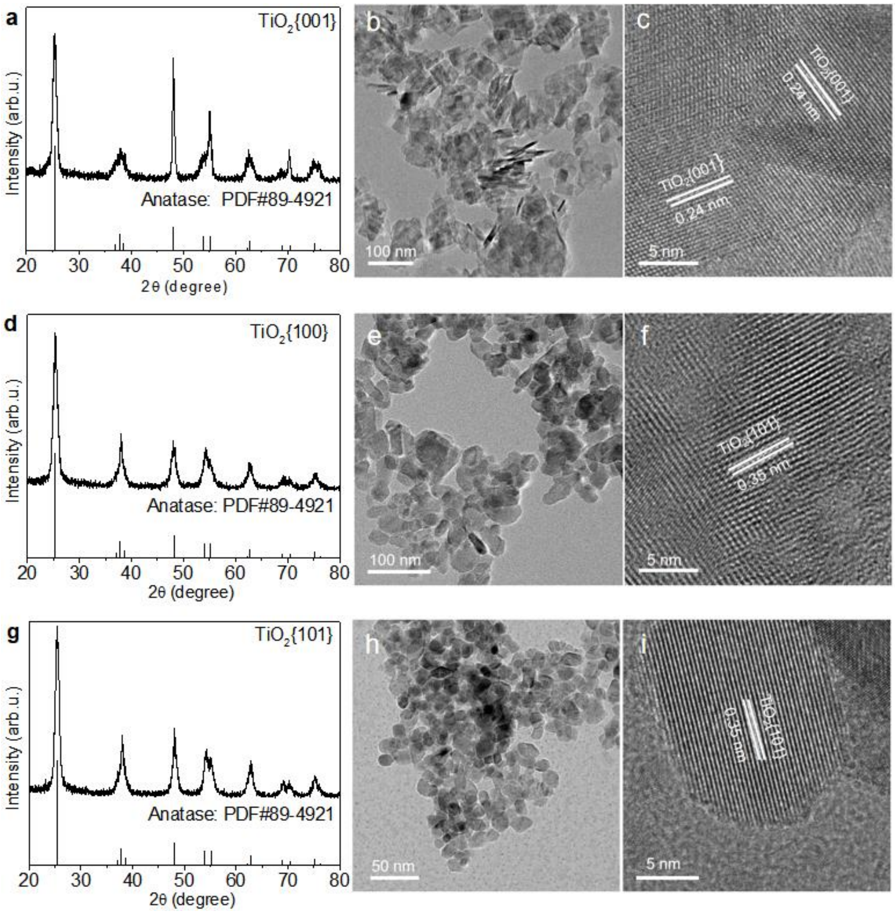

Supplementary Fig. 1. Structural characterizations. XRD patterns with the standard XRD pattern of anatase TiO_2, TEM and HRTEM images of (a-c) TiO_2{001}, (d-f) TiO_2{100} and (g-i) TiO_2{101}NCs. Source data are provided as a Source Data file.

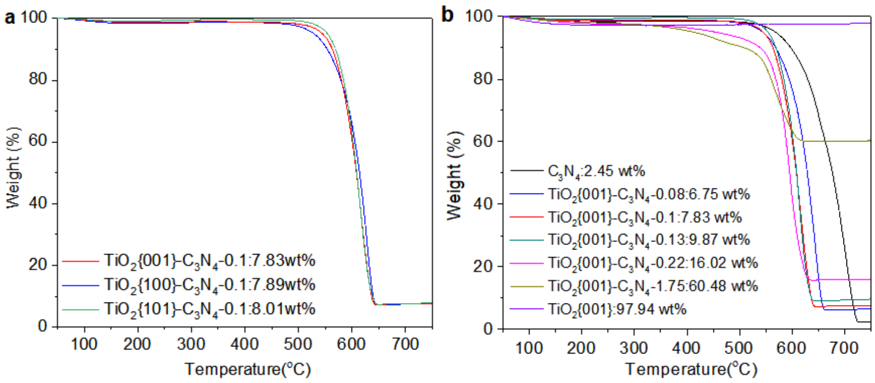

Supplementary Fig. 2. Composition analysis. (a) TGA plots of TiO_2{001}-C_3N_4-0.1, TiO_2{100}-C_3N_4-0.1 and TiO_2{101}-C_3N_4-0.1 composites. (b) TGA plots of various TiO_2{001}-C_3N_4 composites. Source data are provided as a Source Data file.

Supplementary Table 1. Compositions and nomenclature of various TiO_2 NCs-C_3N_4 composites. Source data are provided as a Source Data file.

Mass ratio of precursors	Mass fraction of TiO_2	TiO_2:C_3N_4 mole ratio	Nomenclature of catalysts
TiO_2{001}:$C_2H_4N_4$ = 1:5	60.48%	1.75:1	TiO_2{001}-C_3N_4-1.75
TiO_2{001}:$C_2H_4N_4$ = 1:10	16.02%	0.22:1	TiO_2{001}-C_3N_4-0.22
TiO_2{001}:$C_2H_4N_4$ = 1:30	9.87%	0.13:1	TiO_2{001}-C_3N_4-0.13
TiO_2{001}:$C_2H_4N_4$ = 1:50	7.83%	0.1:1	TiO_2{001}-C_3N_4-0.1
TiO_2{001}:$C_2H_4N_4$ = 1:80	6.75%	0.08:1	TiO_2{001}-C_3N_4-0.08
TiO_2{100}:$C_2H_4N_4$ = 1:50	7.89	0.1:1	TiO_2{100}-C_3N_4-0.1
TiO_2{101}:$C_2H_4N_4$ = 1:50	8.01%	0.1:1	TiO_2{101}-C_3N_4-0.1

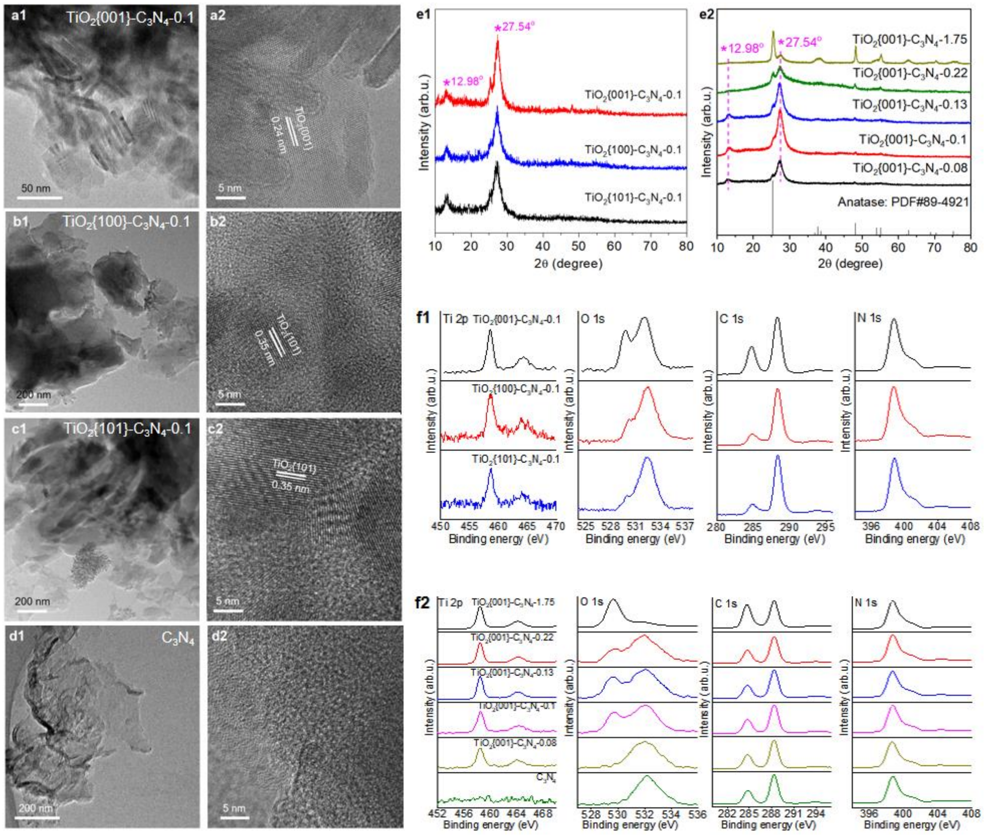

Supplementary Fig. 3. Structural characterizations. TEM and HRTEM images of (a) TiO_2\{001\}-C_3N_4-0.1, (b) TiO_2\{100\}-C_3N_4-0.1, (c) TiO_2\{101\}-C_3N_4-0.1 and (d) C_3N_4. (e) XRD patterns of various TiO_2NCs-C_3N_4 composites. In addition to the diffraction peaks arising from anatase TiO_2, the diffraction peaks of g-C_3N_4 appear at 12.98 and 27.54° respectively corresponding to the repeated tri-s-thiazine (100) units and (002) lattice plane[1,2]. (f) XPS spectra of various TiO_2 NCs-C_3N_4 composites. Source data are provided as a Source Data file.

Supplementary Table 2. Photocatalytic H_2O_2 decomposition over different oxides under the reaction condition of 100% Ar and 10% O_2+90% Ar at 298 K. Photocatalyst amount: 20 mg; reaction time: 30 min; stirring speed: 500 rpm. Source data are provided as a Source Data file.

catalyst	Reaction atmosphere					
	Ar			10% O_2+90% Ar		
	H_2O_2 decomposition		O_2 selectivity	H_2O_2 decomposition		O_2 selectivity
	%	Rate (μmol h^{-1})		%	Rate (μmol h^{-1})	
Blank	1.4%	27.4	99.6%	1.3%	25.4	99.3%
P25	37.3%	730.3	99.6%	26.6%	520.8	99.3%
ZnO	28.8%	563.9	99.3%	18.5%	362.2	99.1%
Fe_2O_3	45.5%	890.9	99.6%	28.1%	550.2	99.1%
WO_3	58.4%	1143.4	99.9%	41.9%	820.4	99.4%
CuO	38.2%	747.9	99.8%	25.6%	501.2	99.1%
V_2O_5	36.4%	712.7	99.4%	27.3%	534.5	99%

Supplementary Table 3. Photocatalytic H_2O_2 decomposition over C_3N_4, TiO_2 NCs and TiO_2 NCs-C_3N_4 composites under the reaction condition of 100% Ar or 10% O_2+90% Ar at 298 K. Photocatalyst amount: 20 mg; reaction time: 30 mins; stirring speed: 500 rpm. Source data are provided as a Source Data file.

catalyst	Reaction atmosphere					
	Ar			10% O_2+90% Ar		
	H_2O_2 decomposition		O_2 selectivity	H_2O_2 decomposition		O_2 selectivity
	%	Rate (μmol h^{-1})		%	Rate (μmol h^{-1})	
C_3N_4	1.9%	37.2	94%	1.8%	35.2	93.4%
TiO_2{001}	31.2%	610.9	93%	15.4%	301.5	91.8%
TiO_2{100}	34.1%	667.6	96%	16.5%	323.1	94.7%
TiO_2{101}	35.8%	700.9	98%	19.6%	383.7	96.8%
TiO_2{001}-C_3N_4-0.1	20.4%	399.4	89%	8.26%	161.7	86.4%
TiO_2{100}-C_3N_4-0.1	22.4%	438.6	92%	10.63%	208.1	89.8%
TiO_2{101}-C_3N_4-0.1	26.1%	511.0	95%	12.24%	239.6	92.4%

Supplementary Table 4. Photocatalytic performance of various photocatalysts in aqueous-phase photocatalytic conversion of methane under the reaction condition of (Entry 1) 8%CH_4+92%Ar with 165 μL H_2O_2 in 20 mL H_2O or (Entry 2) 8%CH_4+4%O_2+88%Ar with 165 μL H_2O_2 in 20 mL H_2O at 298 K. Photocatalyst amount: 20 mg; reaction time: 8 hours; stirring speed: 500 rpm. *NT* indicates "not detected". Source data are provided as a Source Data file.

Catalyst	CH_4 conversion (%)		Product selectivity (%)												H_2O_2 Decomposition (%)		H_2O_2 utilization efficiency (%)	
			CH_3OH		CH_3OOH		CH_3CH_2OH		CH_3COOH		HCOOH		CO and CO_2					
	Entry 1	Entry 2	Entry 1	Entry 2	Entry 1	Entry 2	Entry 1	Entry 2	Entry 1	Entry 2	Entry 1	Entry 2	Entry 1	Entry 2	Entry 1	Entry 2	Entry 1	Entry 2
P25	0.97	1.73	*NT*	*NT*	2.7	2.9	*NT*	*NT*	*NT*	*NT*	*NT*	*NT*	97.3	97.1	100	100	4.64	7.94
ZnO	0.13	0.2	*NT*	*NT*	*NT*	*NT*	*NT*	*NT*	*NT*	*NT*	*NT*	*NT*	100	100	21.3	27.9	8.57	12.1
Fe_2O_3	0.6	0.95	6.9	7.2	1.3	1.7	*NT*	*NT*	*NT*	*NT*	*NT*	*NT*	91.8	91.1	84.8	96.5	7.65	11.3
WO_3	0.5	0.85	*NT*	*NT*	4.1	4.8	*NT*	*NT*	*NT*	*NT*	*NT*	*NT*	95.9	95.2	100	100	5.2	8.2
CuO	0.2	0.3	*NT*	*NT*	*NT*	*NT*	*NT*	*NT*	*NT*	*NT*	*NT*	*NT*	100	100	39	52.8	3	5.1
V_2O_5	0.3	0.5	*NT*	*NT*	*NT*	*NT*	*NT*	*NT*	*NT*	*NT*	*NT*	*NT*	100	100	65.1	74.2	6.31	9.0
TiO_2{001}	1.23	2.17	6.5	7.8	*NT*	*NT*	*NT*	*NT*	*NT*	*NT*	*22.3*	*33.7*	71.2	58.5	100	100	12.1	18.9
TiO_2{100}	1.07	1.6	3.3	5.1	*NT*	*NT*	*NT*	*NT*	*NT*	*NT*	*NT*	*NT*	96.7	94.9	100	100	5.85	8.48
TiO_2{101}	0.64	0.9	*NT*	*NT*	*NT*	*NT*	*NT*	*NT*	*NT*	*NT*	*NT*	*NT*	100	100	100	100	5.48	7.51
TiO_2{001}-C_3N_4-0.1	8.6	16.7	2.2	3.8	13.6	7.6	2.1	0.5	19.4	15.3	56.4	69.8	6.3	3	89	100	53.4	93.3
TiO_2{100}-C_3N_4-0.1	3.2	4.7	13.3	16.4	32.6	37.6	*NT*	*NT*	*NT*	*NT*	*NT*	*NT*	54.1	46	95	100	16.38	24.4
TiO_2{101}-C_3N_4-0.1	1.8	2.6	*NT*	*NT*	12.4	15.3	*NT*	*NT*	*NT*	*NT*	*NT*	*NT*	87.6	84.7	100	100	10.33	14.3

Supplementary Table 5. Photocatalytic performance of various TiO_2 NCs in aqueous-phase photocatalytic conversion of methane under the reaction condition of 8%CH_4+92%Ar+ different amounts of H_2O_2+ 20 mL H_2O at 298 K. Photocatalyst amount: 20 mg; reaction time: 5 hours; stirring speed: 500 rpm. *NT* indicates "not detected". Source data are provided as a Source Data file.

Catalyst	Amount of H_2O_2 (μL)	CH_4 conversion rate (μmol g^{-1} h^{-1})	Product selectivity (%)/formation rate (μmol g^{-1} h^{-1})				Selectivity of liquid-phase products (%)	H_2O_2 utilization efficiency (%)
			CH_3OOH	CH_3OH	HCOOH	CO_x		
TiO_2{001}	22	25	*NT*	22.3%/5.5	20.3%/5.1	57.4%/14.4	42.6	32.3
	55	29.2	*NT*	20.9%/6.1	25.5%/7.4	53.6%/15.6	46.4	27.6
	110	39.5	*NT*	20.4%/8.0	30.4%/12.0	49.2%/19.4	50.8	21.4
	165	50.1	*NT*	12.6%/6.3	36.2%/18.1	51.2%/25.6	48.8	17.7
	220	62.1	*NT*	11.4%/7.1	34.2%/21.2	54.4%/33.8	45.6	10.1
TiO_2{100}	11	16.7	*NT*	33.3%/5.5	*NT*	66.7%/11.1	33.3	24.1
	22	22.7	*NT*	37.5%/8.5	*NT*	62.5%/14.2	37.5	20.7
	55	33.5	*NT*	46.9%/15.7	*NT*	53.1%/17.8	46.9	15.4
	110	45.9	*NT*	25.1%/11.5	*NT*	74.9%/34.4	25.1	10.3
TiO_2{101}	11	8.3	11.3%/0.9	*NT*	*NT*	88.7%/7.4	11.3	22.1
	22	12.4	16.6%/2.1	*NT*	*NT*	83.4%/10.3	16.6	19.3
	55	20.8	22.1%/4.6	*NT*	NT	77.9%/16.2	22.1	14.8
	110	25	10%/2.5	*NT*	*NT*	90%/22.5	10	9.3

Supplementary Table 6. Photocatalytic performance of various TiO_2 NCs in aqueous-phase photocatalytic conversion of methane under the reaction condition of 8%CH_4+different amounts of O_2+balanced Ar+ desired amount of H_2O_2+ 20 mL H_2O at 298 K. Photocatalyst amount: 20 mg; reaction time: 5 hours; stirring speed: 500 rpm. *NT* indicates "not detected". Source data are provided as a Source Data file.

Catalyst	Amount of H_2O_2 (μL)	O_2 Concentration (%)	CH_4 conversion rate (μmol g^{-1} h^{-1})	Product selectivity (%)/formation rate (μmol g^{-1} h^{-1})				Selectivity of liquid-phase products (%)	H_2O_2 utilization efficiency (%)
				CH_3OOH	CH_3OH	HCOOH	CO_x		
TiO_2{001}	110	0	39.5	*NT*	20.4%/8.1	30.4%/12.0	49.2%/19.4	50.8	21.4
		0.8	61.3	*NT*	19.1%/11.7	45.9%/28.1	35%/21.5	65	28.8
		1.6	69.7	*NT*	16.8%/11.7	53.9%/37.6	29.3%/20.4	70.7	32.1
		4	73.2	*NT*	14.2%/10.4	40.4%/29.6	45.4%/33.2	54.6	35.7
		8	78.5	*NT*	13%/10.2	19.9%/15.6	67.1%/52.7	32.9	38.8
TiO_2{100}	55	0	33.5	*NT*	46.9%/15.7	*NT*	53.1%/17.8	46.9	15.4
		0.8	43.7	*NT*	58.5%/25.5	*NT*	41.5%/18.1	58.5	20.2
		1.6	50.3	*NT*	63.4%/31.9	*NT*	36.6%/18.4	63.4	22.3
		4	54.4	*NT*	50.2%/27.3	*NT*	49.8%/27.1	50.2	24.1
		8	58.6	*NT*	45.2%/26.5	*NT*	54.8%/32.1	45.2	25.7
TiO_2{101}	55	0	20.8	22.1%/4.6	*NT*	*NT*	77.9%/16.2	22.1	14.8
		0.8	27.1	27%/7.3	*NT*	*NT*	73%/19.8	27	19.3
		1.6	29.2	27.5%/8.0	*NT*	*NT*	72.5%/21.1	27.5	20.3
		4	35.5	22.9%/8.1	*NT*	*NT*	77.1%/27.4	22.9	25.3
		8	39.1	21.8%/8.5	*NT*	*NT*	78.2%/30.6	21.8	27.7

As shown in Supplementary Table 5 and 6, the optimal reaction condition for photocatalytic methane conversion was 8%CH_4+1.6%O_2+90.4%Ar+ 110 μL H_2O_2+ 20 mL H_2O for TiO_2{001} NCs and 8%CH_4+1.6%O_2+90.4%Ar+ 55 μL H_2O_2+ 20 mL H_2O for TiO_2 {100} and {101} NCs at 298 K, which were employed for other experiments.

Supplementary Table 7. Photocatalytic performance of TiO_2 NCs- C_3N_4-0.1 in aqueous-phase photocatalytic conversion of methane under the reaction condition of 8%CH_4+92%Ar+ different amounts of H_2O_2+ 20 mL H_2O at 298 K. Photocatalyst amount: 20 mg; reaction time: 8 hours; stirring speed: 500 rpm. *NT* indicates "not detected". Source data are provided as a Source Data file.

Catalyst	Amount of H_2O_2 (μL)	CH_4 conversion rate (μmol g^{-1} h^{-1})	Product selectivity (%)/formation rate (μmol g^{-1} h^{-1})						Selectivity of liquid-phase products (%)	H_2O_2 utilization efficiency (%)
			CH_3OOH	CH_3OH	HCOOH	CH_3CH_2OH	CH_3COOH	CO_x		
TiO_2\{001\}-C_3N_4-0.1	55	221.9	17.6%/39.0	4.8%/10.6	20.3%/45.0	6.6%/14.6	3.5%/7.8	11.2%/24.8	88.8	68.3
	110	291.2	13.9%/40.5	4.9%/14.2	37.5%/109.2	0.9%/2.6	8.5%/24.7	8.7%/25.3	91.3	61.6
	165	416.7	13.4%/55.8	4.7%/19.6	56.4%/235.0	1.9%/7.9	17.3%/72.1	6.3%/26.2	93.7	53.4
	220	435.6	14.2%/61.8	5.2%/22.6	41.2%/179.5	0.7%/3.0	11.1%/48.3	27.6%/120.2	72.4	49.7
	275	445.2	12.6%/56.1	5.5%/24.5	27.8%/123.7	0.7%/3.1	9.7%/43.2	43.7%/194.6	56.3	43.1
TiO_2\{100\}-C_3N_4-0.1	22	51.7	22.7%/11.7	33.3%/17.2	*NT*	*NT*	*NT*	44%/22.7	56	55.4
	55	133.4	26.7%/35.6	47.4%/63.2	*NT*	*NT*	*NT*	25.9%/34.5	74.1	51.7
	110	201.2	28.5%/57.3	19.6%/39.4	*NT*	*NT*	*NT*	51.9%/104.4	48.1	46.1
	165	235.3	22.6%/53.2	13.2%/31.1	*NT*	*NT*	*NT*	64.2%/151.1	35.8	36.8
TiO_2\{101\}-C_3N_4-0.1	11	49.1	27.1%/13.3	*NT*	*NT*	*NT*	*NT*	72.9%/35.8	27.1	43.1
	22	75.7	32.9%/24.9	*NT*	*NT*	*NT*	*NT*	67.1%/50.8	32.9	37.3
	55	94.2	22.5%/21.2	*NT*	*NT*	*NT*	*NT*	77.5%/73.0	22.5	28.4
	110	111.9	16.8%/18.8	*NT*	*NT*	*NT*	*NT*	83.2%/93.1	16.8	22.3
	165	121.3	12.4%/15.0	*NT*	*NT*	*NT*	*NT*	87.6%/106.2	12.4	17.7

Supplementary Table 8. Photocatalytic performance of TiO_2 NCs- C_3N_4-0.1 in aqueous-phase photocatalytic conversion of methane under the reaction condition of 8%CH_4+ different amounts of O_2+balanced Ar+ desired amount of H_2O_2+ 20 mL H_2O at 298 K. Photocatalyst amount: 20 mg; reaction time: 8 hours; stirring speed: 500 rpm. *NT* indicates "not detected. Source data are provided as a Source Data file.

Catalyst	Amount of H_2O_2 (μL)	O_2 Concentration (%)	CH_4 conversion rate (μmol g^{-1} h^{-1})	Product selectivity (%)/formation rate (μmol g^{-1} h^{-1})						Selectivity of liquid-phase products (%)	H_2O_2 utilization efficiency (%)
				CH_3OOH	CH_3OH	HCOOH	CH_3CH_2OH	CH_3COOH	CO_x		
TiO_2{001}-C_3N_4-0.1	165	0	416.7	13.4%/55.8	4.7%/19.6	56.4%/235.0	1.9%/7.9	17.3%/72.1	6.3%/26.2	80.6	53.4
		0.8	508.6	8.1%/41.2	3.5%/17.8	63.3%/321.9	1.6%/8.1	17.9%/91.0	5.6%/28.5	94.4	71.1
		1.6	587.7	7.9%/46.4	3.6%/21.1	65.1%/382.6	1.1%/6.4	17.9%/105.2	4.4%/25.8	95.6	82.6
		4	696.3	7.6%/52.9	3.8%/26.4	69.8%/486.0	0.5%/3.5	15.3%/106.5	3%/20.9	97	93.3
		8	829.2	7.1%/58.9	3.7%/30.7	58.8%/487.6	1.3%/10.8	15.1%/125.2	14%/116.1	86	93.5
		12	1067.8	7.3%/77.9	3.5%/37.3	44.1%/470.9	1.4%/14.9	15.8%/168.7	27.9%/297.9	72.1	94.1
TiO_2{100}-C_3N_4-0.1	55	0	133.4	26.7%/35.6	47.4%/63.2	*NT*	*NT*	*NT*	25.9%/34.5	74.1	51.7
		0.8	173.7	31.1%/54.0	45.3%/78.7	*NT*	*NT*	*NT*	23.6%/41.0	76.4	68.8
		1.6	186.8	26.9%/50.2	50.6%/94.5	*NT*	*NT*	*NT*	22.5%/42.0	77.5	70.4
		4	195.9	26.7%/52.3	55.3%/108.3	*NT*	*NT*	*NT*	18%/35.3	82	72.7
		8	221	27.8%/61.4	41.7%/92.1	*NT*	*NT*	*NT*	30.5%/67.4	69.5	78.1
		12	241.8	26.3%/63.6	32.5%/78.6	*NT*	*NT*	*NT*	41.2%/99.6	58.8	84.7
TiO_2{101}-C_3N_4-0.1	22	0	75.7	32.9%/24.9	*NT*	*NT*	*NT*	*NT*	67.1%/50.8	32.9	37.3
		0.8	91.1	34.3%/31.2	*NT*	*NT*	*NT*	*NT*	65.7%/59.8	34.3	48.2
		1.6	99.2	36.1%/35.8	*NT*	*NT*	*NT*	*NT*	63.9%/63.4	36.1	49.6
		4	108.4	37.7%/40.8	*NT*	*NT*	*NT*	*NT*	62.3%/67.5	37.7	51.3
		8	121.3	25.9%/31.4	*NT*	*NT*	*NT*	*NT*	74.1%/89.9	25.9	61.7
		12	136.2	11.1%/15.1	*NT*	*NT*	*NT*	*NT*	88.9%/121.1	11.1	64.4

As shown in Supplementary Table 7 and 8, the optimal reaction condition for photocatalytic methane conversion was 8%CH_4+4%O_2+88%Ar+ 165μL H_2O_2+ 20 mL H_2O for TiO_2{001}-C_3N_4-0.1, 8%CH_4+4%O_2+88%Ar+ 55 μL H_2O_2+ 20 mL H_2O for TiO_2{100}-C_3N_4-0.1 and 8%CH_4+4%O_2+88%Ar+ 22 μL H_2O_2+ 20 mL H_2O for TiO_2{101}-C_3N_4-0.1 at 298 K, which were employed for other experiments.

Supplementary Table 9. Photocatalytic performance of TiO_2 {001}-C_3N_4 composites with different TiO_2:C_3N_4 molar ratios in aqueous-phase photocatalytic conversion of methane under the reaction condition of 8%CH_4+ 4%O_2+88% Ar+ 165 µL H_2O_2+ 20 mL H_2O at 298 K. Photocatalyst amount: 20 mg; reaction time: 8 hours; stirring speed: 500 rpm. *NT* indicates "not detected". Source data are provided as a Source Data file.

Catalyst	CH_4 conversion rate (µmol g^{-1} h^{-1})	Product selectivity (%)/formation rate (µmol g^{-1} h^{-1})						Selectivity of liquid-phase products (%)
		CH_3OOH	CH_3OH	HCOOH	CH_3CH_2OH	CH_3COOH	CO_x	
TiO_2 {001}-C_3N_4-0.08	479.2	11%/52.7	5.1%/24.4	65.3%/312.9	0.1%/0.5	12.7%/60.8	5.8%/27.8	94.2
TiO_2 {001}-C_3N_4-0.1	696.3	7.6%/52.9	3.8%/26.4	69.8%/486.0	0.5%/3.5	15.3%/106.5	3%/20.9	97
TiO_2 {001}-C_3N_4-0.13	546.5	*NT*	4.9%/26.8	60.7%/331.7	0.4%/2.2	17%/92.9	17%/92.9	83
TiO_2 {001}-C_3N_4-0.22	168.8	*NT*	6.6%/11.1	12.9%/21.8	9.3%/15.7	16.4%/27.7	54.8%/92.5	45.2
TiO_2 {001}-C_3N_4-1.75	145.3	*NT*	12.5%/18.2	16.3%/23.7	0.9%/1.3	14.7%/21.3	55.6%/80.8	44.4

Supplementary Table 10. Photocatalytic performance of various photocatalysts in aqueous-phase photocatalytic conversion of methane under the optimal reaction conditions for different reaction times at 298 K. Photocatalyst amount: 20 mg; stirring speed: 500 rpm. *NT* indicates "not detected". Source data are provided as a Source Data file.

Catalyst	Reaction condition	Time	CH_4 conversion rate (μmol g^{-1} h^{-1})	Product selectivity (%)/formation rate (μmol g^{-1} h^{-1})						Selectivity of liquid-phase products (%)
				CH_3OOH	CH_3OH	HCOOH	CH_3CH_2OH	CH_3COOH	CO_x	
TiO_2 {001}	8% CH_4 +1.6% O_2 +90.4%Ar +110 μL H_2O_2 +20 mL H_2O	2h	23.1	*NT*	14.9%/3.4	50.3%/11.6	*NT*	*NT*	34.8%/8.0	65.2
		5h	69.7	*NT*	16.8%/11.7	53.9%/37.6	*NT*	*NT*	29.3%/20.4	70.7
		8h	84.3	*NT*	6.4%/5.4	31.7%/26.7	*NT*	*NT*	61.9%/52.2	38.1
TiO_2 {100}	8% CH_4 +1.6% O_2 +90.4%Ar +55 μL H_2O_2 +20 mL H_2O	2h	17.4	*NT*	59.9%/10.4	*NT*	*NT*	*NT*	40.1%/7.0	59.9
		5h	50.3	*NT*	63.4%/31.9	*NT*	*NT*	*NT*	36.6%/18.4	63.4
		8h	78.2	*NT*	29.1%/22.7	*NT*	*NT*	*NT*	70.9%/55.4	29.1
TiO_2 {101}	8% CH_4 +1.6% O_2 +90.4%Ar +55 μL H_2O_2 +20 mL H_2O	2h	9.7	28.4%/2.7	*NT*	*NT*	*NT*	*NT*	71.6%/6.9	28.4
		5h	29.2	27.5%/8.0	*NT*	*NT*	*NT*	*NT*	72.5%/21.2	27.5
		8h	40.2	11.8%/4.7	*NT*	*NT*	*NT*	*NT*	88.2%/35.4	11.8
TiO_2 {001}-C_3N_4-0.1	8% CH_4 +4% O_2 +90.4%Ar+ 165 μL H_2O_2 +20 mL H_2O	5h	335.2	*NT*	3.9%/13.1	63.8%/213.8	0.9%/3.0	18.6%/62.3	12.8%/42.9	87.2
		8h	696.3	7.6%/52.9	3.8%/26.4	69.8%/486.0	0.5%/3.5	15.3%/106.5	3%/20.9	97
		12h	736.8	*NT*	*NT*	67%/493.6	0.7%/5.1	20.4%/150.3	11.9%/87.7	88.1
TiO_2 {100}-C_3N_4-0.1	8% CH_4 +1.6% O_2 +90.4%Ar +55 μL H_2O_2 +20 mL H_2O	5h	137.4	36.3%/49.8	47.9%/65.8	*NT*	*NT*	*NT*	15.8%/21.7	84.2
		8h	195.9	26.7%/52.3	55.3%/108.3	*NT*	*NT*	*NT*	18%/35.3	82
		12h	258.3	7.3%/18.8	38.5%/99.4	*NT*	*NT*	*NT*	54.2%/140.0	45.8
TiO_2 {101}-C_3N_4-0.1	8% CH_4 +1.6% O_2 +90.4%Ar +22 μL H_2O_2 +20 mL H_2O	5h	71.9	41.9%/30.1	*NT*	*NT*	*NT*	*NT*	58.1%/41.8	41.9
		8h	108.4	37.7%/40.8	*NT*	*NT*	*NT*	*NT*	62.3%/67.5	37.7
		12h	158.3	18.4%/29.1	*NT*	*NT*	*NT*	*NT*	81.6%/129.1	18.4

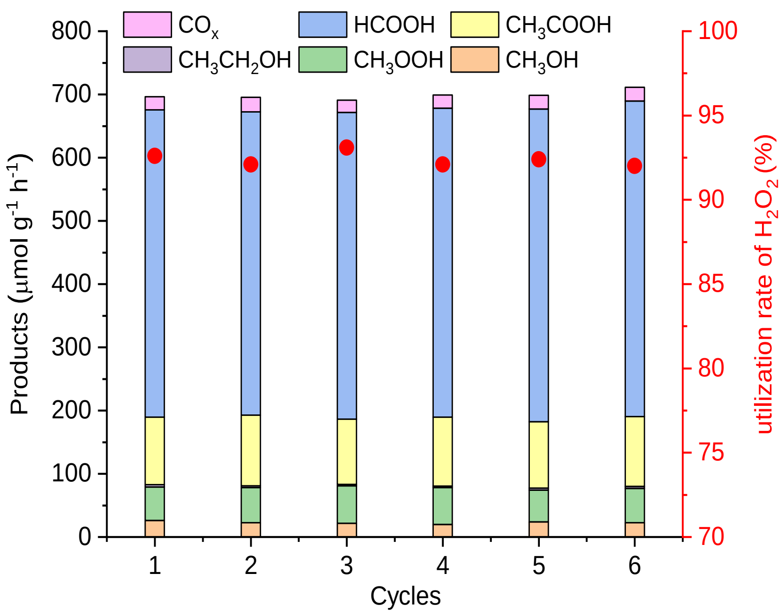

Supplementary Fig. 4. Photocatalytic performance. Photocatalytic performance of TiO_2\{001\}-C_3N_4-0.1 for six cycles under the reaction condition of 8%CH_4+4%O_2+88%Ar+165 μL H_2O_2+20 mL H_2O at 298 K. Photocatalyst amount: 20 mg; reaction time: 8 hours; stirring speed: 500 rpm. Source data are provided as a Source Data file.

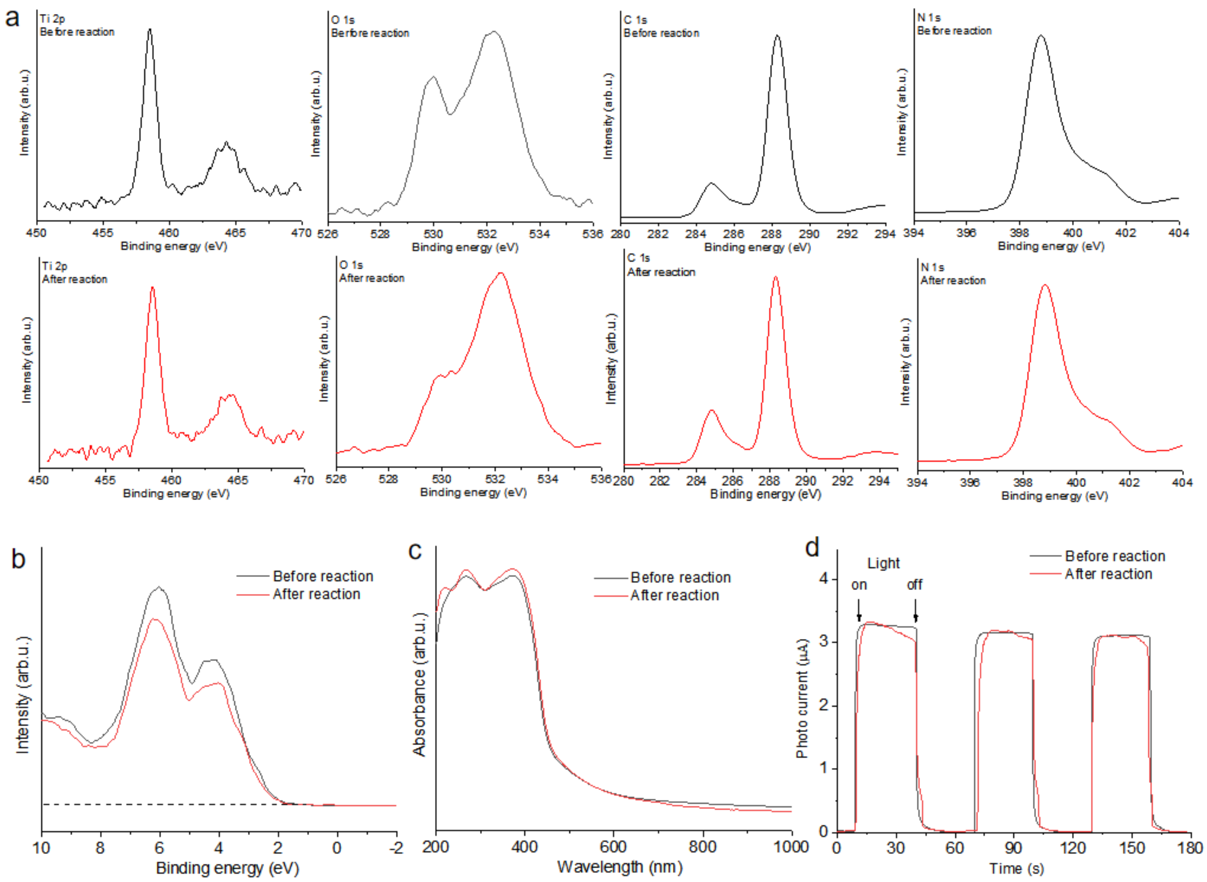

Supplementary Fig. 5. Structural characterizations. (a) XPS, (b) VB-XPS, (c) PL and (d) photocurrent measurements of TiO_2 {001}-C_3N_4-0.1 before and after six cycles of photocatalytic performance evaluation under the reaction condition of 8%CH_4+4%O_2+88%Ar+165 μL H_2O_2+20 mL H_2O at 298 K (photocatalyst amount: 20 mg; reaction time: 8 hours; stirring speed: 500 rpm). Source data are provided as a Source Data file.

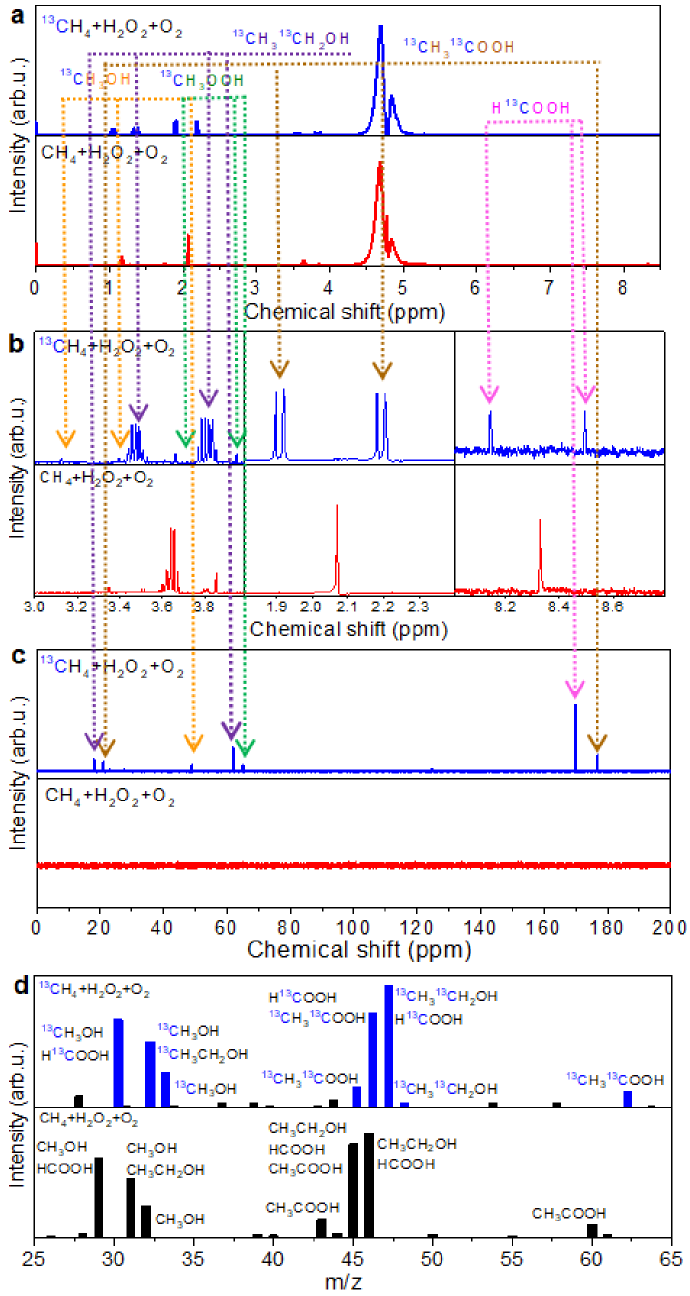

Supplementary Fig. 6. Product analysis. (a,b) ^{1}H NMR spectra of liquid-phase products of aqueous-phase photocatalytic conversion of methane over TiO_2\{001\}-C_3N_4-0.1 under the reaction condition of 4%O_2+88%Ar+165 μL H_2O_2+20 mL H_2O and of 8%CH_4+4%O_2+88%Ar+165 μL H_2O_2+20 mL H_2O at 298 K using CH_4 or $^{13}CH_4$ at 298 K.The splitting peaks due to ^{1}H-^{13}C J coupling for $^{13}CH_3OH$, $^{13}CH_3OOH$, $H^{13}COOH$, $^{13}CH_3{}^{13}CH_2OH$, and $^{13}CH_3{}^{13}COOH$ observed in these ^{1}H NMR spectra. (c) ^{13}C with decoupling NMR spectra of liquid-phase products of aqueous-phase photocatalytic conversion of methane over TiO_2\{001\}-C_3N_4-0.1 under the reaction condition of 4%O_2+88%Ar+165 μL H_2O_2+20 mL H_2O and of 8%CH_4+4%O_2+88%Ar+165 μL H_2O_2+20 mL H_2O at 298 K using CH_4 or $^{13}CH_4$ at 298 K. (d) MS spectra of liquid-phase products of aqueous-phase photocatalytic conversion of methane over TiO_2\{001\}-C_3N_4-0.1 under the reaction condition of 8%CH_4+4%O_2+88%Ar+165 μL H_2O_2+20 mL H_2O at 298 K using $^{13}CH_4$ (top panel)or CH_4 (bottom panel) at 298 K. Photocatalyst amount: 20 mg; reaction time: 8 hours; stirring speed: 500 rpm. Source data are provided as a Source Data file.

Supplementary Table 11. Photocatalytic performance of TiO_2\{001\}-C_3N_4-0.1 in aqueous-phase photocatalytic conversion of methane for different reaction times at 298 K. Photocatalyst amount: 20 mg; stirring speed: 500 rpm. Reaction condition: 8%CH_4+4%O_2+88%Ar+ 165 µL H_2O_2+20 mL H_2O; *NT* indicates "not detected". Source data are provided as a Source Data file.

Reaction times	CH_4 conversion rate (µmol g^{-1} h^{-1})	Products (µmol)							Selectivity of liquid-phase oxygenates (%)
		CH_3OOH	CH_3OH	HCHO	HCOOH	CH_3CH_2OH	CH_3COOH	CO_x	
10min	27.3	0.05	0.02	0.01	*NT*	*NT*	*NT*	0.007	95.5
30min	38.4	0.1	0.08	0.02	0.15	0.01	*NT*	0.01	95.4
1h	83.1	0.06	0.37	*NT*	0.89	0.02	0.08	0.13	92

Supplementary Table 12. Photocatalytic performance of $TiO_2(001)$ and $TiO_2\{001\}$-C_3N_4-0.1 in aqueous-phase photocatalytic conversion of methane under the optimal reaction conditions at 298 K using O_2 and $^{18}O_2$. Photocatalyst amount: 20 mg; stirring speed: 500 rpm. *NT* indicates "not detected". Reaction condition for $TiO_2(001)$: 8%CH_4+1.6%O_2+90.4%Ar+110 μL H_2O_2+20 mL H_2O; time: 5 h. Reaction condition for $TiO_2\{001\}$-C_3N_4-0.1: 8%CH_4+4%O_2+88%Ar+ 165 μL H_2O_2+20 mL H_2O; time: 8 h. Source data are provided as a Source Data file.

Catalyst	oxygen	CH_4 conversion rate (μmol g^{-1} h^{-1})	Product selectivity (%)						Selectivity of liquid-phase products (%)	H_2O_2 Decomposition (%)	H_2O_2 utilization efficiency (%)
			CH_3OH	CH_3OOH	CH_3CH_2OH	CH_3COOH	HCOOH	CO_x			
$TiO_2\{001\}$	O_2	69.7	16.8	*NT*	*NT*	*NT*	53.9	29.3	70.7	100	32.1
	$^{18}O_2$	61.2	18.3	*NT*	*NT*	*NT*	49.5	32.2	67.8	100	30.7
$TiO_2\{001\}$-C_3N_4-0.1	O_2	696.3	3.8	7.6	0.5	15.3	69.8	3	97	100	93.3
	$^{18}O_2$	675.7	3.1	6.9	0.3	18.9	66.6	4.2	95.8	100	91.8

As described in the Materials & Methods section, when O_2 was used, $n_{O2\ reacted}$ was calculated from the amount of products and the ratio of the products formed by O_2 based on the isotope-labelling results for the calculations of H_2O_2 utilization efficiency, and when $^{18}O_2$ was used, $n_{18O2\ reacted}$ was calculated by $(n(^{18}O_2)_{before\ reaction}$-$n(^{18}O_2)_{after\ reaction})$, in which $n(^{18}O_2)$ was quantified using GC-MS (as shown above). It can be seen that the data calculated by both methods were almost the same.

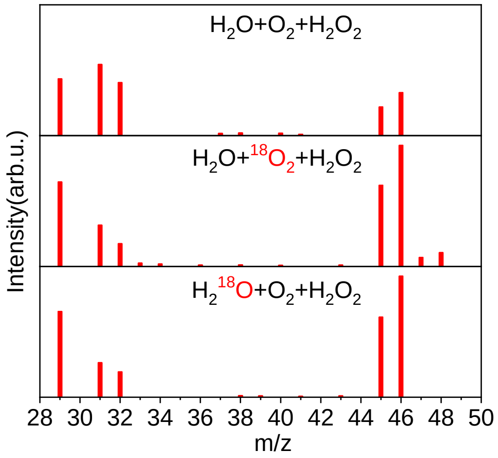

Supplementary Fig. 7. Product analysis. As-measured mass spectra of liquid-phase products of aqueous-phase photocatalytic conversion of methane over TiO_2\{001\} NCs under the reaction condition of 8%CH_4+1.6%O_2+90.4%Ar+110 μL H_2O_2+20 mL H_2O at 298 K using $H_2O+O_2+H_2O_2$, $H_2O+^{18}O_2+H_2O_2$, or $H_2^{18}O+O_2+H_2O_2$. Photocatalyst amount: 20 mg; reaction time: 5 hours; stirring speed: 500 rpm. Source data are provided as a Source Data file.

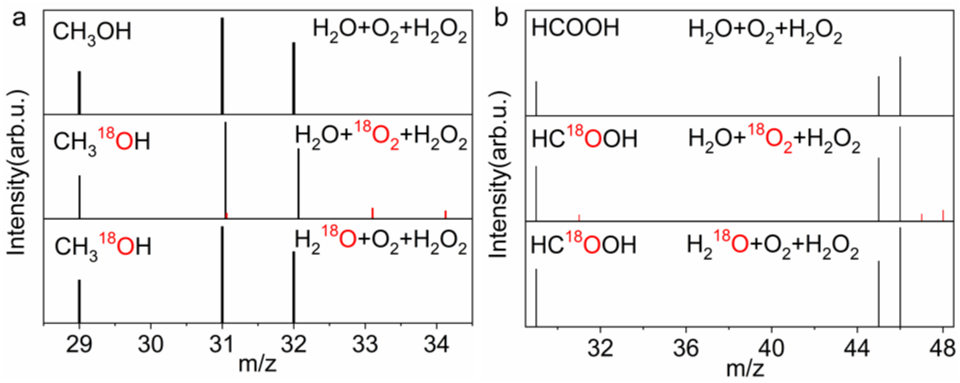

Supplementary Fig. 8. Product analysis. Mass spectra of (a) methanol and (b) formic acid formed during aqueous-phase photocatalytic conversion of methane over TiO_2\{001\} NCs under the reaction condition of 8%CH_4+1.6%O_2+90.4% Ar+110 μL H_2O_2+20 mL H_2O at 298 K using $H_2O+O_2+H_2O_2$, $H_2O+{}^{18}O_2+H_2O_2$, or $H_2{}^{18}O+O_2+H_2O_2$. These spectra were derived from those in Supplementary Figure 7 by subtracting the contributions of fragments based on the NIST standard mass spectra of methanol (https://webbook.nist.gov/cgi/cbook.cgi?ID=C67561&Units=SI&Mask=200#Mass-Spec) and formic acid (https://webbook.nist.gov/cgi/cbook.cgi?ID=C64186&Units=SI&Mask=200#Mass-Spec) as the following:

CH_3OH: $I_{32\text{-}CH3OH}=I_{32\text{-}peak}$; $I_{31\text{-}CH3OH}=I_{32\text{-}CH3OH}/0.744$; $I_{29\text{-}CH3OH}=I_{31\text{-}CH3OH}*0.446$

$CH_3{}^{18}OH$: $I_{34\text{-}CH318OH}=I_{34\text{-}peak}$; $I_{33\text{-}CH318OH}=I_{34\text{-}CH318OH}/0.744$; $I_{31\text{-}CH318OH}=I_{33\text{-}CH318OH}*0.446$

HCOOH: $I_{46\text{-}HCOOH}=I_{46\text{-}peak}$; $I_{45\text{-}HCOOH}=I_{46\text{-}HCOOH}*0.664$; $I_{29\text{-}HCOOH}=I_{46\text{-}HCOOH}*0.582$

$HC^{18}OOH$: $I_{48\text{-}HC18OOH}=I_{48\text{-}peak}$; $I_{47\text{-}HC18OOH}=I_{48\text{-}HC18OOH}*0.664$; $I_{31\text{-}HC18OOH}=I_{48\text{-}HC18OOH}*0.582$

The reliability of our analysis was proved by comparing $I_{29\text{-}peak}$ with ($I_{29\text{-}CH3OH}+I_{29\text{-}HCOOH}$) and $I_{31\text{-}peak}$ with ($I_{31\text{-}CH3OH}+I_{31\text{-}CH318OH}+I_{31\text{-}HC18OOH}$). The error between $I_{29\text{-}peak}$ and ($I_{29\text{-}CH3OH}+I_{29\text{-}HCOOH}$) was 1%, 0.7% and 1.13% for the cases of $H_2O+O_2+H_2O_2$, $H_2O+{}^{18}O_2+H_2O_2$, or $H_2{}^{18}O+O_2+H_2O_2$, respectively, and the error between $I_{31\text{-}peak}$ and ($I_{31\text{-}CH3OH}+I_{31\text{-}CH318OH}+I_{31\text{-}HC18OOH}$) was 0.73% for the case of $H_2O+{}^{18}O_2+H_2O_2$. Source data are provided as a Source Data file.

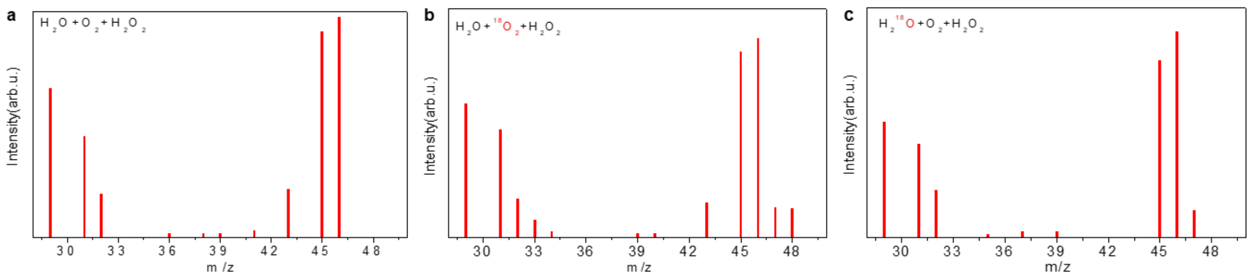

Supplementary Fig. 9. Product analysis. As-measured mass spectra of liquid-phase products of aqueous-phase photocatalytic conversion of methane over $TiO_2\{001\}$-C_3N_4-0.1 under the reaction condition of $8\%CH_4+4\%O_2+88\%$ Ar+165 μL H_2O_2+20 mL H_2O at 298 K using (a) $H_2O+O_2+H_2O_2$, (b) $H_2O+^{18}O_2+H_2O_2$, or (c) $H_2{}^{18}O+O_2+H_2O_2$. Photocatalyst amount: 20 mg; reaction time: 8 hours; stirring speed: 500 rpm. Mass spectra of methanol, formic acid, ethanol and acetic acid shown in Figure 2 a-d were derived from these spectra by subtracting the contributions of fragments based on the NIST standard mass spectra of methanol (https://webbook.nist.gov/cgi/cbook.cgi?ID=C67561&Units=SI&Mask=200#Mass-Spec), formic acid (https://webbook.nist.gov/cgi/cbook.cgi?ID=C64186&Units=SI&Mask=200#Mass-Spec), ethanol (https://webbook.nist.gov/cgi/cbook.cgi?ID=C64175&Units=SI&Mask=200#Mass-Spec) and acetic acid (https://webbook.nist.gov/cgi/cbook.cgi?ID=C64197&Units=SI&Mask=200#Mass-Spec) as the following:

CH_3OH: $I_{32-CH3OH}=I_{32-peak}$; $I_{31-CH3OH}=I_{32-CH3OH}/0.744$; $I_{29-CH3OH}=I_{31-CH3OH}*0.446$

$CH_3{}^{18}OH$: $I_{34-CH318OH}=I_{34-peak}$; $I_{33-CH318OH}=I_{34-CH318OH}/0.744$; $I_{31-CH318OH}=I_{33-CH318OH}*0.446$

$HCOOH$: $I_{29-HCOOH}=I_{29-peak}-I_{29-CH3OH}$; $I_{46-HCOOH}=I_{29-HCOOH}/0.582$; $I_{45-HCOOH}=I_{46-HCOOH}*0.664$

CH_3CH_2OH: $I_{46-CH3CH2OH}=I_{46-peak}-I_{46-HCOOH}$; $I_{31-CH3CH2OH}=I_{46-CH3CH2OH}/0.246$; $I_{45-CH3CH2OH}=I_{31-CH3CH2OH}*0.573$

$HC^{18}OOH$: $I_{31-HC18OOH}=I_{31-peak}-I_{31-CH3OH}-I_{31-CH318OH}-I_{31-CH3CH2OH}$; $I_{48-HC18OOH}=I_{31-HC18OOH}/0.582$; $I_{47-HC18OOH}=I_{48-HC18OOH}*0.664$

$CH_3CH_2{}^{18}OH$: $I_{33-CH3CH218OH}=I_{33-peak}-I_{33-CH3CH2OH}$; $I_{47-CH3CH218OH}=I_{33-CH3CH218OH}*0.573$; $I_{48-CH3CH218OH}=I_{33-CH3CH218OH}*0.246$

$CH_3C^{18}O^{18}OH$: $I_{49-CH3C18O18OH}=I_{49-peak}$; $I_{45-CH3C18O18OH}=I_{49-CH3C18O18OH}/0.9$

$CH_3CO^{18}OH$: $I_{47-CH3CO18OH}=I_{47-peak}-I_{47-HC18OOH}-I_{47-CH3CH218OH}$; $I_{43-CH3CO18OH}=I_{47-CH3CO18OH}/0.9$

CH_3COOH: $I_{43-CH3COOH}=I_{43-peak}-I_{43-CH3CO18OH}$; $I_{45-CH3COOH}=I_{43-CH3COOH}*0.9$

The reliability of our analysis was proved by comparing $I_{45-peak}$ with ($I_{45-HCOOH}+I_{45-CH3CH2OH}+I_{45-CH3C18O18OH}+I_{45-CH3COOH}$). The error between $I_{45-peak}$ and ($I_{45-HCOOH}+I_{45-CH3CH2OH}+I_{45-CH3C18O18OH}+I_{45-CH3COOH}$) was 0.68%, 1.13% and 0.55% for the cases of $H_2O+O_2+H_2O_2$, $H_2O+^{18}O_2+H_2O_2$, or $H_2{}^{18}O+O_2+H_2O_2$, respectively. Source data are provided as a Source Data file.

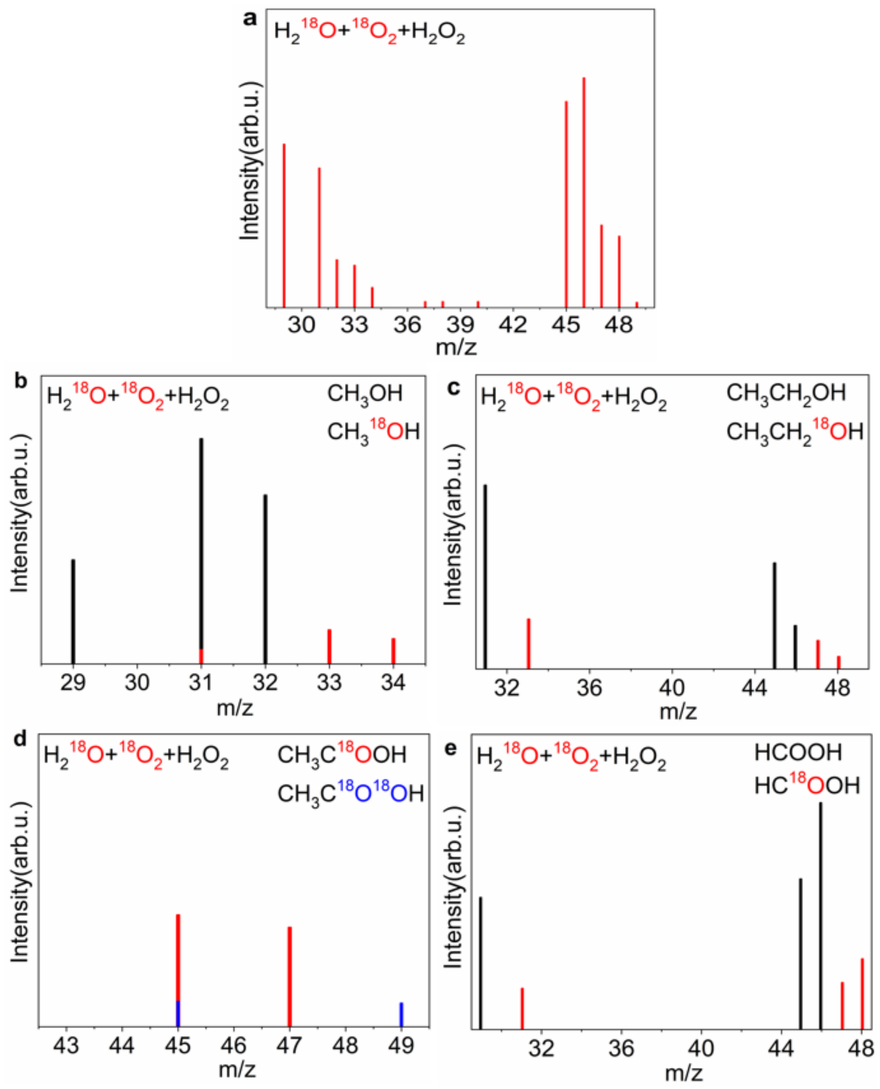

Supplementary Fig. 10. Product analysis. (a) As-measured mass spectrum of liquid-phase products of aqueous-phase photocatalytic conversion of methane over TiO_2\{001\}-C_3N_4-0.1 under the reaction condition of 8%CH_4+4%$^{18}O_2$+88% Ar+165 µL H_2O_2+20 mL $H_2{}^{18}O$ at 298 K. Photocatalyst amount: 20 mg; reaction time: 8 hours; stirring speed: 500 rpm. Mass spectra of (b) methanol, (c) formic acid, (d) ethanol and (e) acetic acid derived from the as-measured mass spectrum in Panel (a) by subtracting the contributions of fragments based on the NIST standard mass spectra of methanol (https://webbook.nist.gov/cgi/cbook.cgi?ID=C67561&Units=SI&Mask=200#Mass-Spec), formic acid (https://webbook.nist.gov/cgi/cbook.cgi?ID=C64186&Units=SI&Mask=200#Mass-Spec), ethanol (https://webbook.nist.gov/cgi/cbook.cgi?ID=C64175&Units=SI&Mask=200#Mass-Spec) and acetic acid (https://webbook.nist.gov/cgi/cbook.cgi?ID=C64197&Units=SI&Mask=200#Mass-Spec) as described in the caption of Supplementary Figure 9.

The reliability of our analysis was proved by comparing $I_{45\text{-peak}}$ with ($I_{45\text{-HCOOH}}$+ $I_{45\text{-CH3CH2OH}}$+ $I_{45\text{-CH3C18O18OH}}$+ $I_{45\text{-CH3COOH}}$). The error between $I_{45\text{-peak}}$ and ($I_{45\text{-HCOOH}}$+ $I_{45\text{-CH3CH2OH}}$+ $I_{45\text{-CH3C18O18OH}}$+ $I_{45\text{-CH3COOH}}$) was 0.47% for the case of $H_2{}^{18}O+{}^{18}O_2+H_2O_2$. Source data are provided as a Source Data file.

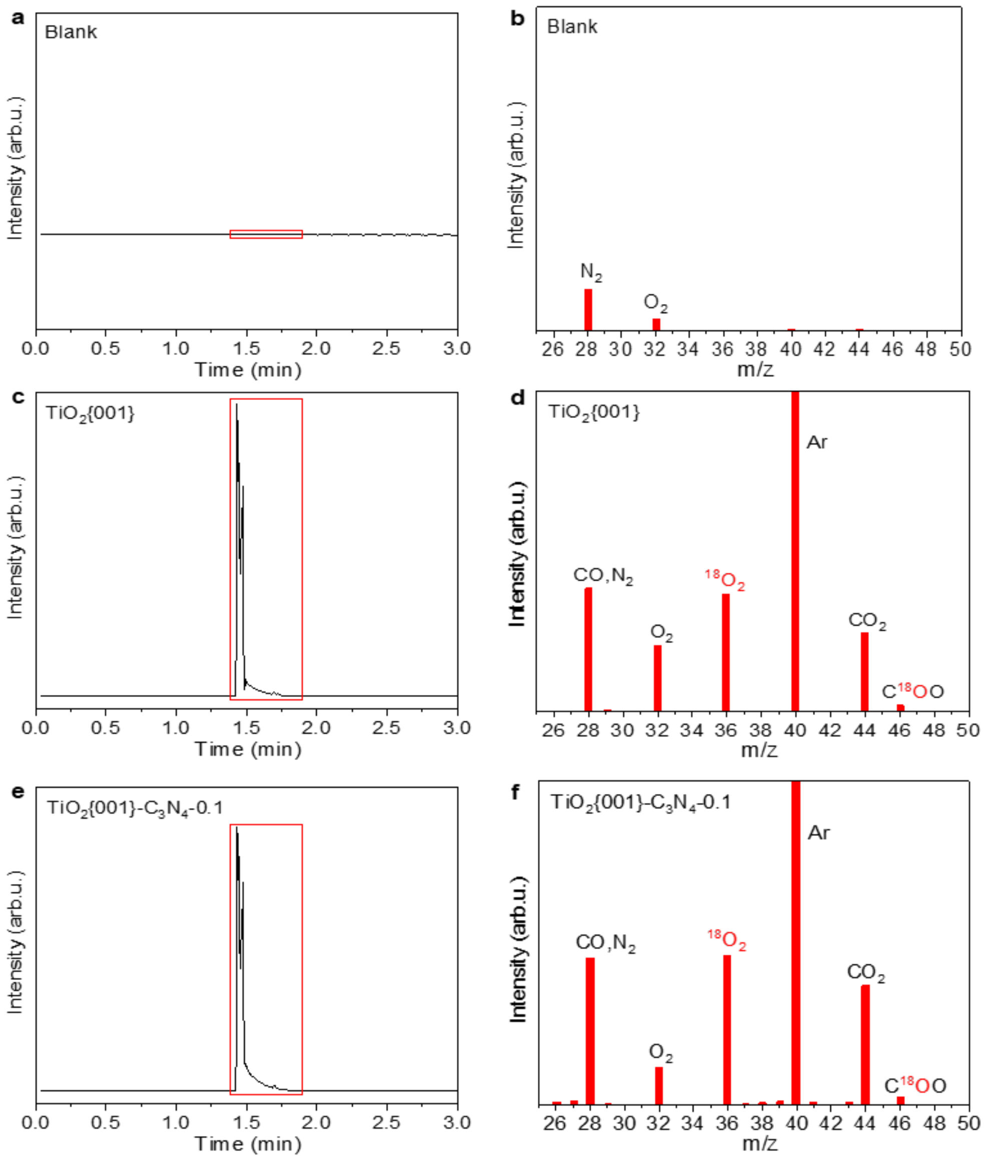

Supplementary Fig. 11. Product analysis. GC-MS spectra of (a and b) blank test, gas-phase products of aqueous-phase photocatalytic conversion of methane (c and d) over TiO_2 {001} NCs under the reaction condition of 8% CH_4 +1.6% O_2 +90.4% Ar+110 μL H_2O_2 +20 mL H_2O at 298 K using H_2O + $^{18}O_2$ + H_2O_2 (Photocatalyst amount: 20 mg; reaction time: 5 hours; stirring speed: 500 rpm), and (e and f) over TiO_2 {001}- C_3N_4 -0.1 under the reaction condition of 8% CH_4 +4% O_2 +88% Ar+165 μL H_2O_2 +20 mL H_2O at 298 K using H_2O + $^{18}O_2$ + H_2O_2 (Photocatalyst amount: 20 mg; reaction time: 8 hours; stirring speed: 500 rpm). The HP-5MS column in the used Trace GC/ISQ MS was not able to separate CO, N_2, O_2, Ar and CO_2, and the mass spectra were measured over across the time range indicated in the GC spectra by the red rectangle. Source data are provided as a Source Data file.

Supplementary Table 13. Photocatalytic performance of TiO_2 {001}-C_3N_4-0.1 in aqueous-phase photocatalytic conversion of methane under the optimal reaction conditions at 298 K using $^{18}O_2$. Photocatalyst amount: 20 mg; stirring speed: 500 rpm. *NT* indicates "not detected". Reaction condition for TiO_2 {001}-C_3N_4-0.1: 8%CH_4+12%O_2+80%Ar+ 165 µL H_2O_2+20 mL H_2O; time: 8 h. Source data are provided as a Source Data file.

Catalyst	oxygen	CH_4 conversion rate (µmol g^{-1} h^{-1})	Product selectivity (%)						Selectivity of liquid-phase products (%)	H_2O_2 Decomposition (%)	H_2O_2 utilization efficiency (%)
			CH_3OH	CH_3OOH	CH_3CH_2OH	CH_3COOH	HCOOH	CO_x			
TiO_2 {001}-	O_2	1067.8	3.5	7.3	1.4	15.8	44.1	27.9	72.1	100	94.1
C_3N_4-0.1	$^{18}O_2$	989.7	2.7	8.1	1.1	14.4	42.9	30.8	67.2	100	93.3

As described in the Materials & Methods section, when O_2 was used, $n_{O2\ reacted}$ was calculated from the amount of products and the ratio of the products formed by O_2 based on the isotope-labelling results for the calculations of H_2O_2 utilization efficiency, and when $^{18}O_2$ was used, $n_{18O2\ reacted}$ was calculated by $(n(^{18}O_2)_{before\ reaction}$-$n(^{18}O_2)_{after\ reaction})$, in which $n(^{18}O_2)$ was quantified using GC-MS (as shown above). It can be seen that the data calculated by both methods were almost the same.

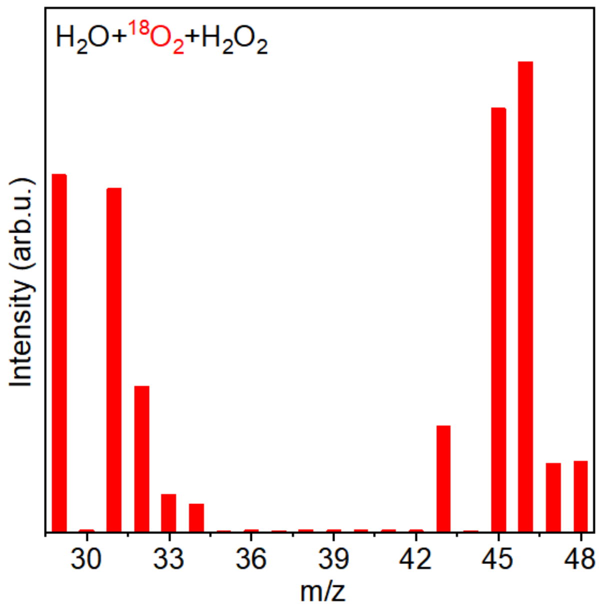

Supplementary Fig. 12. Product analysis. As-measured mass spectra of liquid-phase products of aqueous-phase photocatalytic conversion of methane over TiO_2{001}-C_3N_4-0.1 under the reaction condition of 8%CH_4+12%O_2+80% Ar+165 μL H_2O_2+20 mL H_2O at 298 K using H_2O+$^{18}O_2$+H_2O_2. Photocatalyst amount: 20 mg; reaction time: 8 hours; stirring speed: 500 rpm. Source data are provided as a Source Data file.

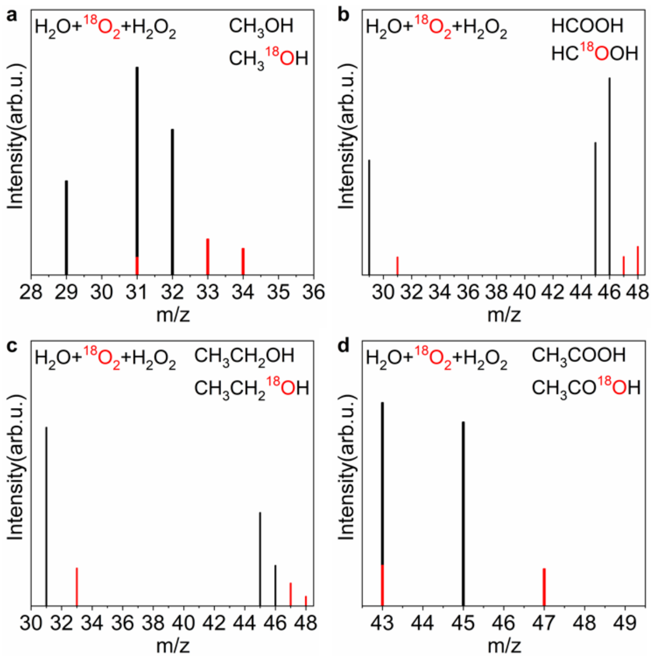

Supplementary Fig. 13. Product analysis. Mass spectra of (a) methanol, (b) formic acid, (c) ethanol and (d) acetic acid during aqueous-phase photocatalytic conversion of methane over $TiO_2\{001\}$-C_3N_4-0.1 under the reaction condition of 8%CH_4+12%O_2+80% Ar+165 μL H_2O_2+20 mL H_2O at 298 K using $H_2O+{}^{18}O_2+H_2O_2$ derived from Supplementary Figure 12 by subtracting the contributions of fragments based on the NIST standard mass spectra of methanol (https://webbook.nist.gov/cgi/cbook.cgi?ID=C67561&Units=SI&Mask=200#Mass-Spec), formic acid (https://webbook.nist.gov/cgi/cbook.cgi?ID=C64186&Units=SI&Mask=200#Mass-Spec), ethanol (https://webbook.nist.gov/cgi/cbook.cgi?ID=C64175&Units=SI&Mask=200#Mass-Spec) and acetic acid (https://webbook.nist.gov/cgi/cbook.cgi?ID=C64197&Units=SI&Mask=200#Mass-Spec) as the following:

CH_3OH: $I_{32\text{-}CH3OH}=I_{32\text{-}peak}$; $I_{31\text{-}CH3OH}=I_{32\text{-}CH3OH}/0.744$; $I_{29\text{-}CH3OH}=I_{31\text{-}CH3OH}*0.446$

$CH_3{}^{18}OH$: $I_{34\text{-}CH318OH}=I_{34\text{-}peak}$; $I_{33\text{-}CH318OH}=I_{34\text{-}CH318OH}/0.744$; $I_{31\text{-}CH318OH}=I_{33\text{-}CH318OH}*0.446$

$HCOOH$: $I_{29\text{-}HCOOH}=I_{29\text{-}peak}-I_{29\text{-}CH3OH}$; $I_{46\text{-}HCOOH}=I_{29\text{-}HCOOH}/0.582$; $I_{45\text{-}HCOOH}= I_{46\text{-}HCOOH}*0.664$

CH_3CH_2OH: $I_{46\text{-}CH3CH2OH}=I_{46\text{-}peak}-I_{46\text{-}HCOOH}$; $I_{31\text{-}CH3CH2OH}=I_{46\text{-}CH3CH2OH}/0.246$; $I_{45\text{-}CH3CH2OH}=I_{31\text{-}CH3CH2OH}*0.573$

$HC^{18}OOH$: $I_{31\text{-}HC18OOH}=I_{31\text{-}peak}-I_{31\text{-}CH3OH}-I_{31\text{-}CH318OH}-I_{31\text{-}CH3CH2OH}$; $I_{48\text{-}HC18OOH}=I_{31\text{-}HC18OOH}/0.582$; $I_{47\text{-}HC18OOH}=I_{48\text{-}HC18OOH}*0.664$

$CH_3CH_2{}^{18}OH$: $I_{33\text{-}CH3CH218OH}=I_{33\text{-}peak}-I_{33\text{-}CH3CH2OH}$; $I_{47\text{-}CH3CH218OH}=I_{33\text{-}CH3CH218OH}*0.573$; $I_{48\text{-}CH3CH218OH}=I_{33\text{-}CH3CH218OH}*0.246$

$CH_3CO^{18}OH$: $I_{47\text{-}CH3CO18OH}=I_{47\text{-}peak}-I_{47\text{-}HC18OOH}-I_{47\text{-}CH3CH218OH}$; $I_{43\text{-}CH3CO18OH}=I_{47\text{-}CH3CO18OH}/0.9$

CH_3COOH: $I_{43\text{-}CH3COOH}=I_{43\text{-}peak}-I_{43\text{-}CH3CO18OH}$; $I_{45\text{-}CH3COOH}=I_{43\text{-}CH3COOH}*0.9$

The reliability of our analysis was proved by comparing $I_{45\text{-}peak}$ with ($I_{45\text{-}HCOOH}+ I_{45\text{-}CH3CH2OH}+ I_{45\text{-}CH3C18O18OH}+ I_{45\text{-}CH3COOH}$). The error between $I_{45\text{-}peak}$ and ($I_{45\text{-}HCOOH}+ I_{45\text{-}CH3CH2OH}+ I_{45\text{-}CH3C18O18OH}+ I_{45\text{-}CH3COOH}$) was 1.49% for the cases of $H_2O+{}^{18}O_2+H_2O_2$. Source data are provided as a Source Data file.

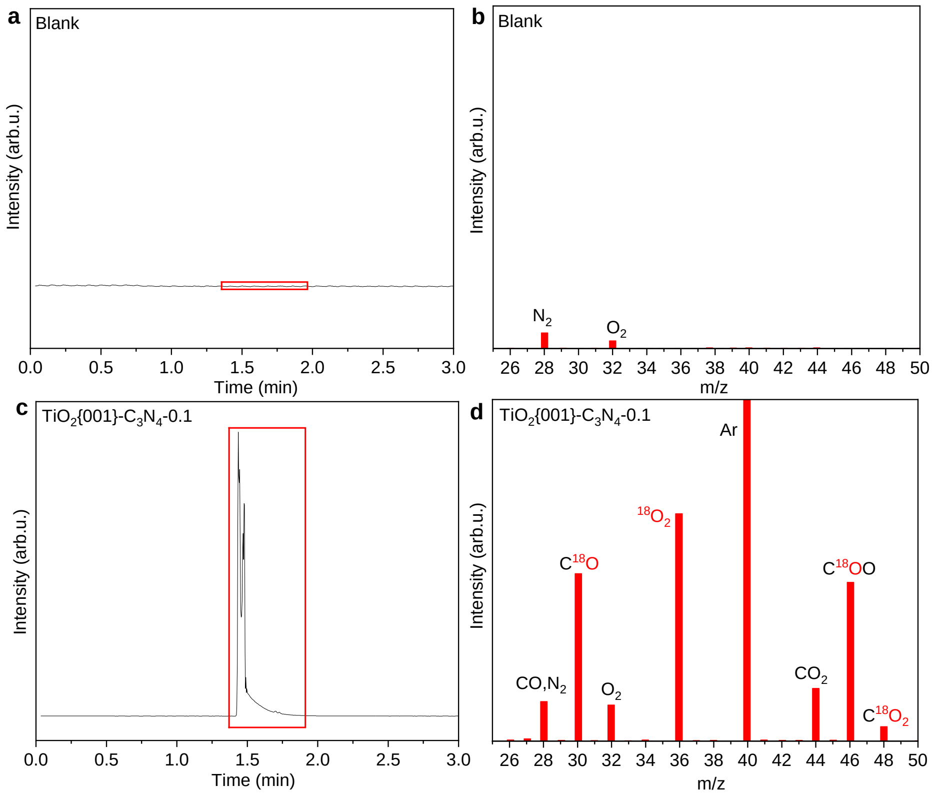

Supplementary Fig. 14. Product analysis. GC-MS spectra of (a and b) blank test, (c and d) gas-phase products of aqueous-phase photocatalytic conversion of methane over $TiO_2\{001\}$-C_3N_4-0.1 under the reaction condition of 8%CH_4+12%O_2+80% Ar+165 μL H_2O_2+20 mL H_2O at 298 K using H_2O+$^{18}O_2$+H_2O_2 (Photocatalyst amount: 20 mg; reaction time: 8 hours; stirring speed: 500 rpm). The HP-5MS column in the used Trace GC/ISQ MS was not able to separate CO, N_2, O_2, Ar and CO_2, and the mass spectra were measured over across the time range indicated in the GC spectra by the red rectangle. Source data are provided as a Source Data file.

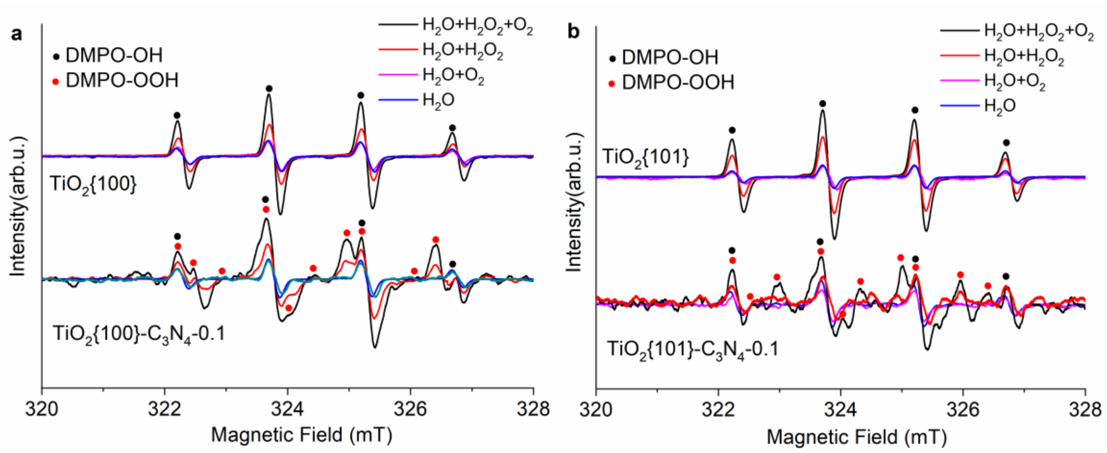

Supplementary Fig. 15. Radical intermediate characterizations. In situ ESR spectra of H_2O (5 mL H_2O+3μL DMPO), H_2O+O_2 (O_2 +5 mL H_2O+3μL DMPO), $H_2O+H_2O_2$ (10μL H_2O_2+5 mL H_2O+3μL DMPO) and $H_2O+O_2+H_2O_2$ (O_2+10μL H_2O_2+5 mL H_2O+3μL DMPO) solutions under UV light illumination in the presence of DMPO over (a) TiO_2{100} NCs and TiO_2{100}-C_3N_4-0.1 composites and (b) TiO_2{101} NCs and TiO_2{101}-C_3N_4-0.1 composites at 298 K. All spectra were taken after 2 min's UV light illumination. Photocatalyst amount: 2.5 mg. Source data are provided as a Source Data file.

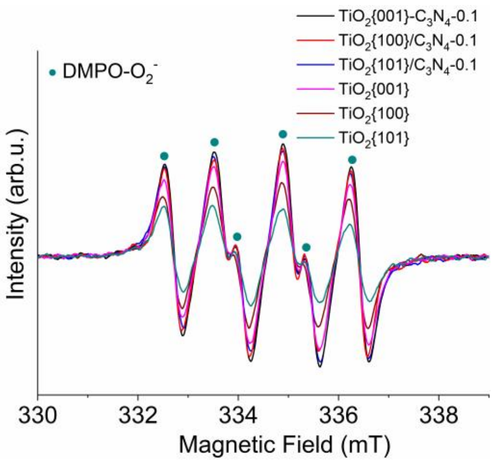

Supplementary Fig. 16. Radical intermediate characterizations. In situ ESR spectra of O_2 in methanol (O_2 +5 mL CH_3OH+3μL DMPO) under UV light illumination in the presence of DMPO over TiO_2 NCs and TiO_2-C_3N_4 composites at 298 K. All spectra were taken after 2 min's UV light illumination. Photocatalyst amount: 2.5 mg. Source data are provided as a Source Data file.

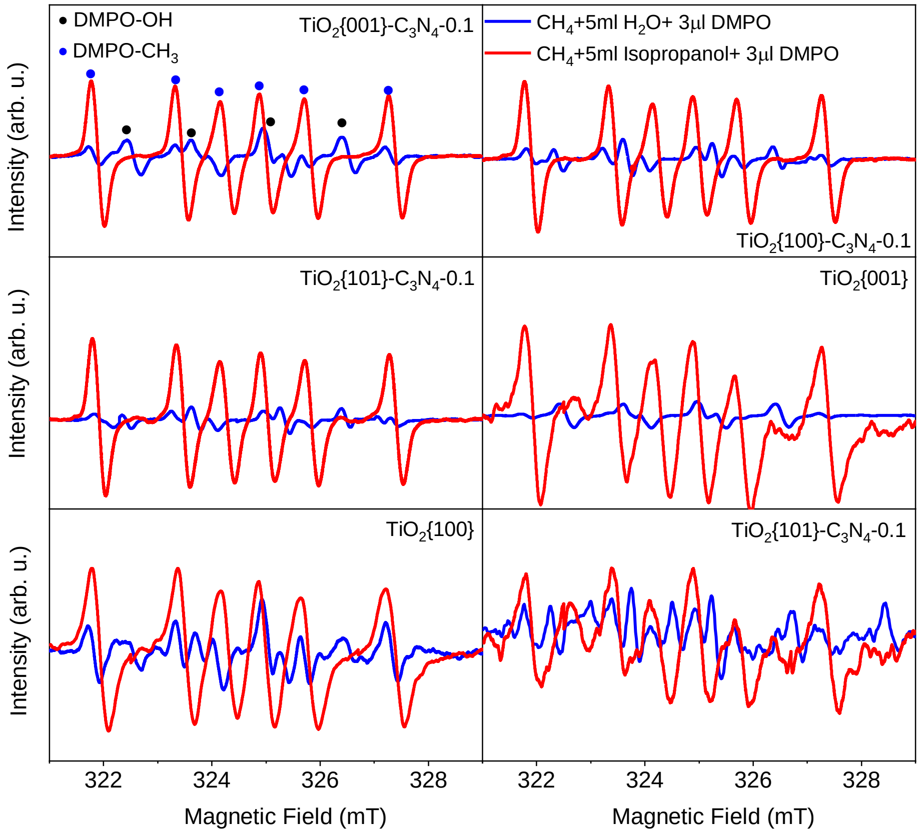

Supplementary Fig. 17. Radical intermediate characterizations. In situ ESR spectra of CH_4+H_2O mixture (CH_4 +5 mL H_2O+3μL DMPO) and CH_4+isopropyl alcohol mixture (CH_4 +5 mL isopropyl alcohol+3μL DMPO) under UV light illumination in the presence of DMPO over TiO_2 NCs and TiO_2 NCs-C_3N_4-0.1 composites at 298 K. All spectra were taken after 2 min' UV light illumination. Photocatalyst amount: 2.5 mg. Source data are provided as a Source Data file.

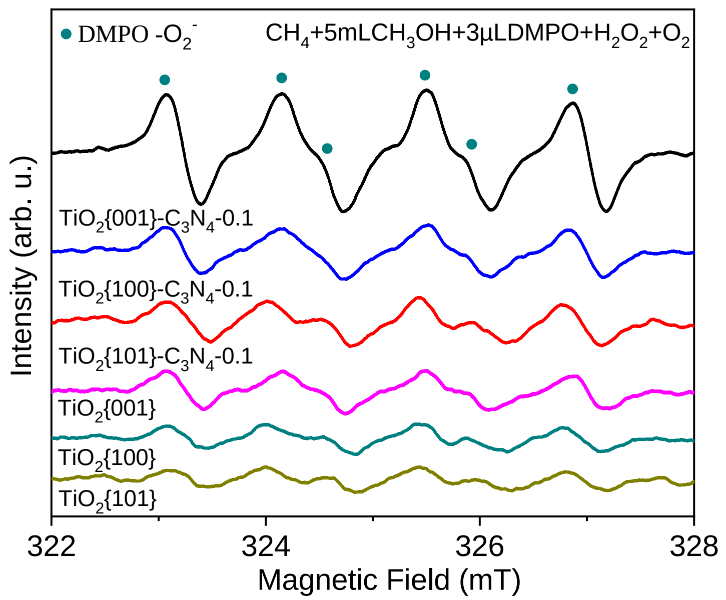

Supplementary Fig. 18. Radical intermediate characterizations. In situ ESR spectra of CH_4+methanol mixture (CH_4+5 mL CH_3OH+3μL DMPO)+H_2O_2+O_2 under UV light illumination in the presence of DMPO over TiO_2 NCs and TiO_2 NCs-C_3N_4-0.1 composites at 298 K. All spectra were taken after 2 min's UV light illumination. Photocatalyst amount: 2.5 mg. Source data are provided as a Source Data file.

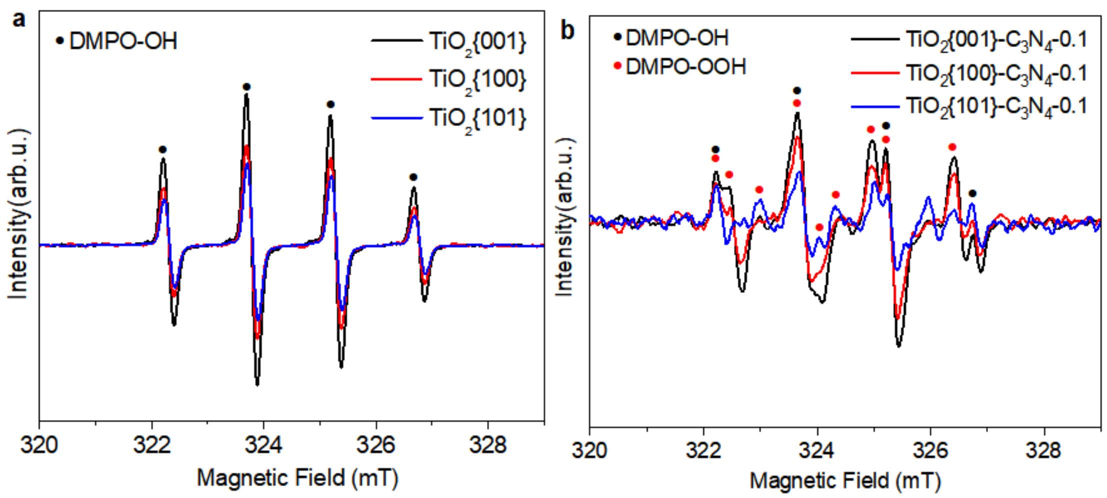

Supplementary Fig. 19. Radical intermediate characterizations. In situ ESR spectra of H_2O+H_2O_2 mixture (10μL H_2O_2+5 mL H_2O+3μL DMPO) under UV light illumination in the presence of DMPO over (a) TiO_2 NCs and (b) TiO_2 NCs-C_3N_4-0.1 composites at 298 K. All spectra were taken after 2 min's UV light illumination. Photocatalyst amount: 2.5 mg. Source data are provided as a Source Data file.

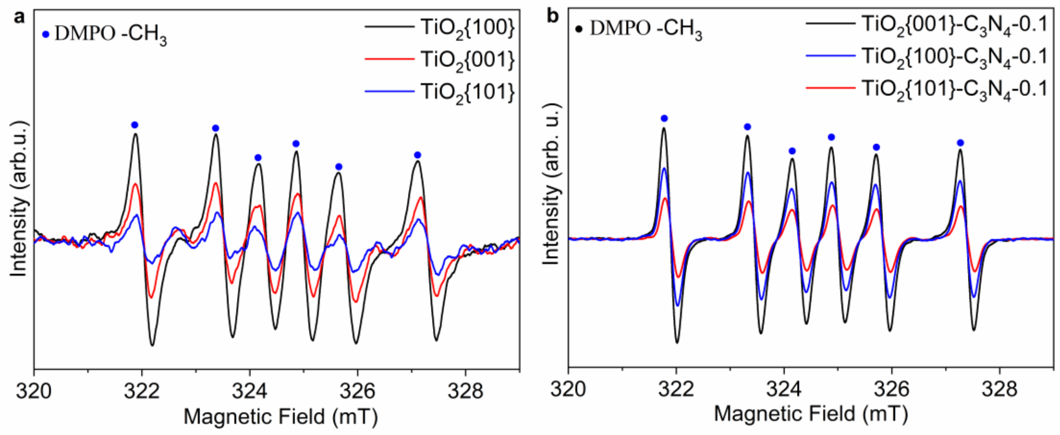

Supplementary Fig. 20. Radical intermediate characterizations. In situ ESR spectra of CH_4+isopropyl alcohol mixture (CH_4 +5 mL isopropyl alcohol+3μL DMPO) under UV light illumination in the presence of DMPO over (a) TiO_2 NCs and (b) TiO_2 NCs-C_3N_4-0.1 composites at 298 K. All spectra were taken after 2 min's UV light illumination. Photocatalyst amount: 2.5 mg. Source data are provided as a Source Data file.

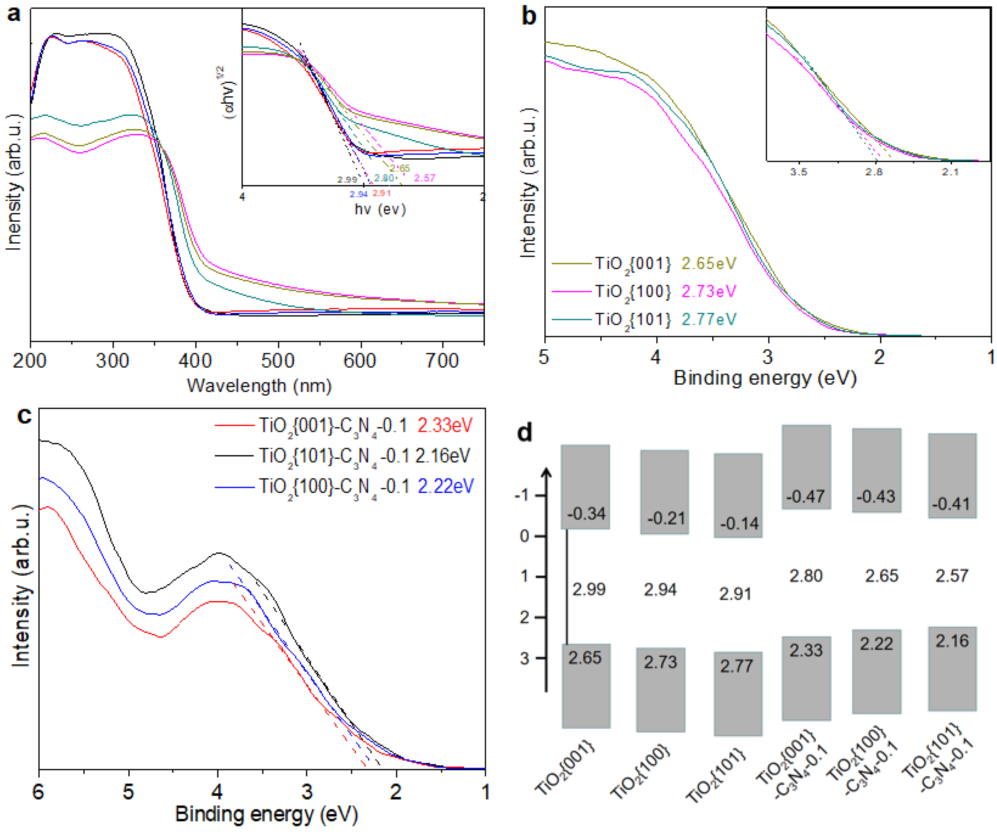

Supplementary Fig. 21. Band structures. (a) UV-vis DRS spectra of TiO_2 NCs and TiO_2 NCs-C_3N_4-0.1 composites. The inset shows calculated band gaps. Valence-band XPS spectra with indicated valence band edges of (b) TiO_2 NCs and (c) TiO_2 NCs-C_3N_4-0.1 composites. (d) Band structures of TiO_2 NCs and TiO_2 NCs-C_3N_4-0.1 composites derived from UV-vis DRS spectra (band gap) and valence-band XPS spectra (valence band edge). Source data are provided as a Source Data file.

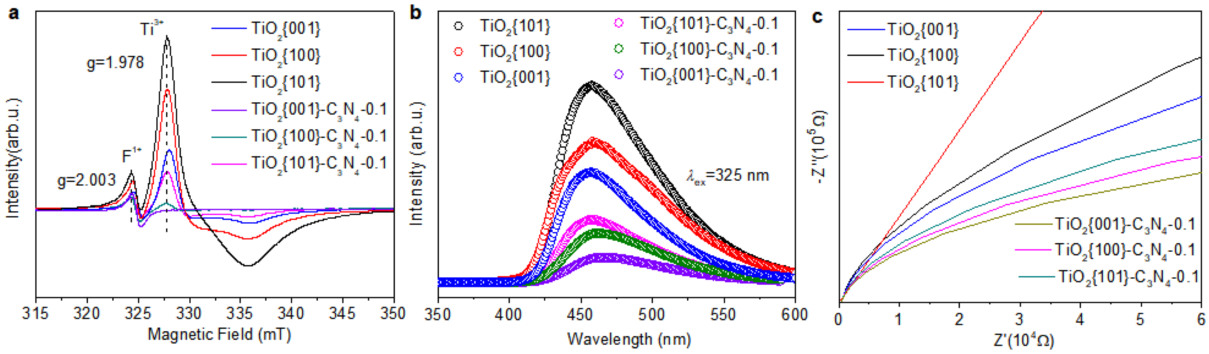

Supplementary Fig. 22. Structural characterizations. (a) EPR spectra, (b) EIS and (c) PL spectra of TiO_2 NCs and TiO_2 NCs-C_3N_4-0.1 composites. Source data are provided as a Source Data file.

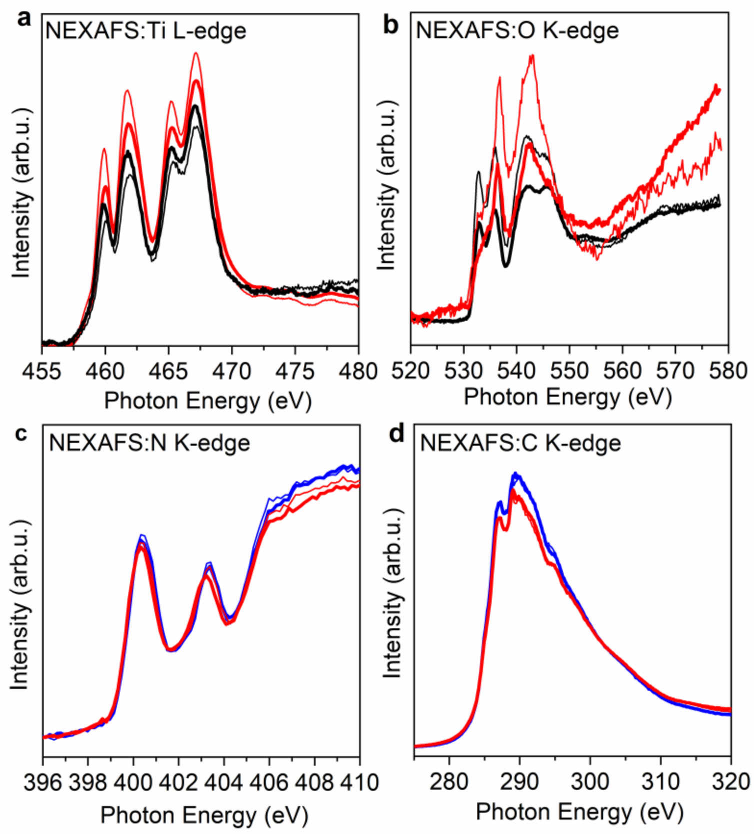

Supplementary Fig. 23. NEXAFS characterizations. (a) Ti L-edge, (b) O K-edge, (c) N K-edge and (d) C K-edge NEXAFS spectra of TiO_2 {100} NCs (black line), TiO_2 {100} NCs-C_3N_4-0.1 composite (red line) and C_3N_4 (blue line) in dark (thick line) and under UV light illumination (thin line). Source data are provided as a Source Data file.

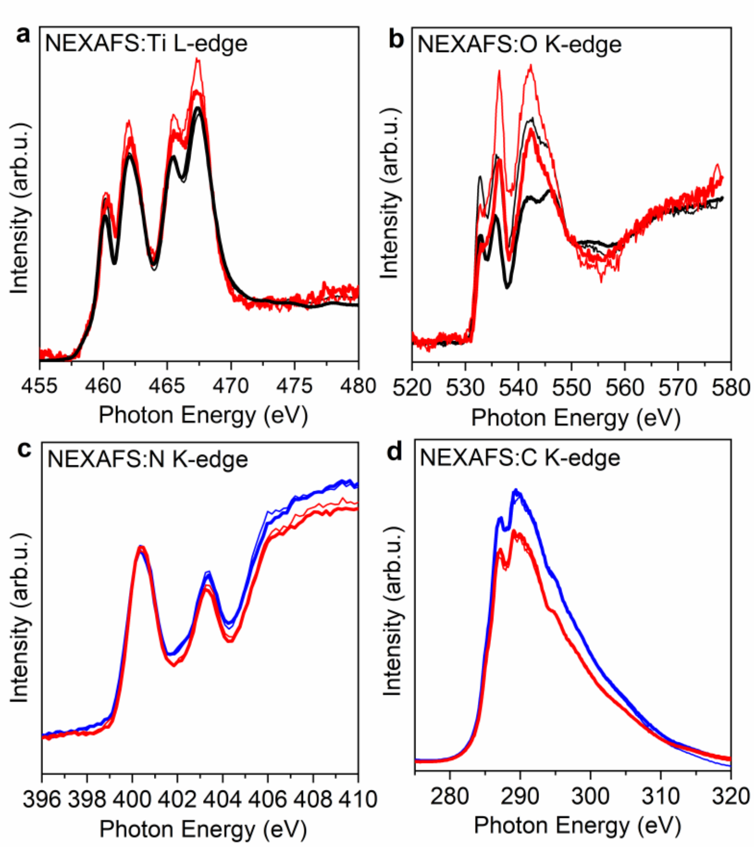

Supplementary Fig. 24. NEXAFS characterizations. (a) Ti L-edge, (b) O K-edge, (c) N K-edge and (d) C K-edge NEXAFS spectra of TiO_2\{101\} NCs (black line), TiO_2\{101\} NCs-C_3N_4-0.1 composite (red line) and C_3N_4 (blue line) in dark (thick line) and under UV light illumination (thin line). Source data are provided as a Source Data file.

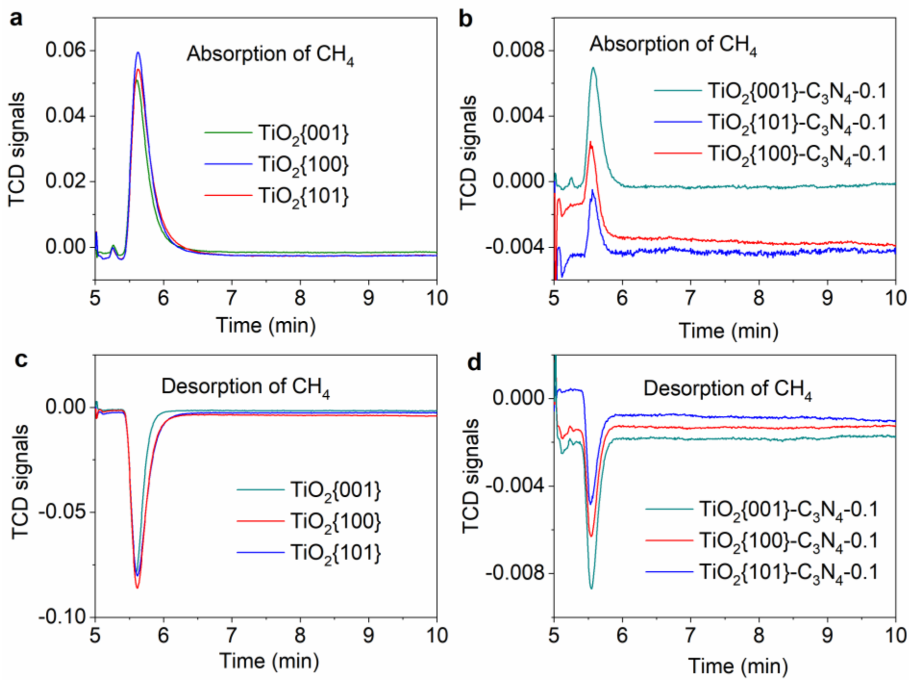

Supplementary Fig. 25. CH_4 adsorption and desorption. TCD signals of adsorption and desorption processes of CH_4 on (a and c) TiO_2 NCs and (b and d) TiO_2 NCs-C_3N_4-0.1 composites at -100 °C. Source data are provided as a Source Data file.

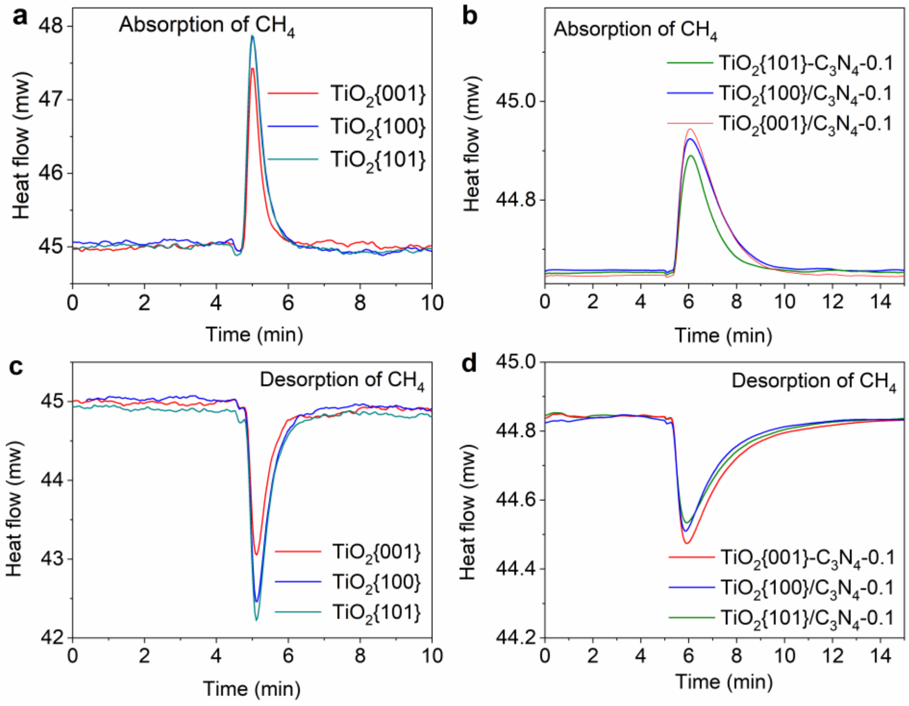

Supplementary Fig. 26. CH_4 adsorption and desorption. Heat flow of adsorption and desorption processes of CH_4 on (a and c) TiO_2 NCs and (b and d) TiO_2 NCs-C_3N_4-0.1 composites at -100 °C. Source data are provided as a Source Data file.

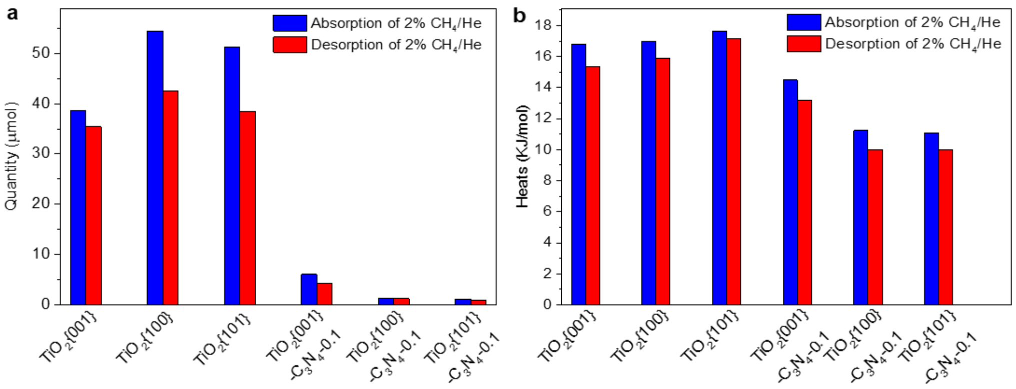

Supplementary Fig. 27. CH_4 adsorption and desorption. Calculated quantity (a) and heats (b) of CH_4 adsorption and desorption on TiO_2 NCs and TiO_2 NCs-C_3N_4-0.1 based on the results shown in Supplementary Figs. 25 and 26. Source data are provided as a Source Data file.

Supplementary Table 14. Assignments of vibrational bands observed in the in situ DRIFTS spectra shown in Figure 4.

Assignment	Wavenumber (cm^{-1})
CH_3	1473 cm^{-1}
CH_2	1445 cm^{-1}
CH_3OH	1019 and 1092 cm^{-1}
CH_3O	1042 and 1156 cm^{-1}
CH_2O	1712 cm^{-1}
HCOO	1526, 1556 and 1564 cm^{-1}
HCOOH	1664 cm^{-1}
Carbonates	1504 and 1592 cm^{-1}
gaseous HCOOH	1760 and 1782 cm^{-1}
gaseous CH_4	1304 cm^{-1}
gaseous CO	2135 and 2170 cm^{-1}
gaseous CO_2	2340 and 2360 cm^{-1}

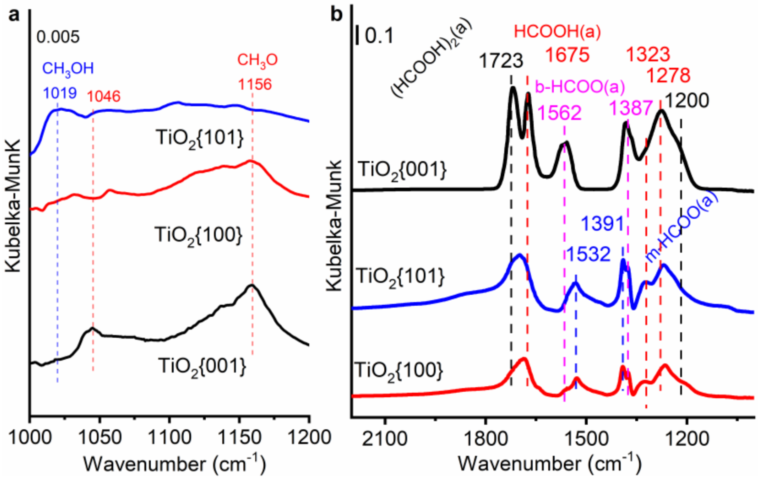

Supplementary Fig. 28. **Methanol and formic acid adsorption.** DRIFTS spectra of (a) methanol and (b) formic acid adsorption on TiO_2 NCs at 298 K. Source data are provided as a Source Data file.

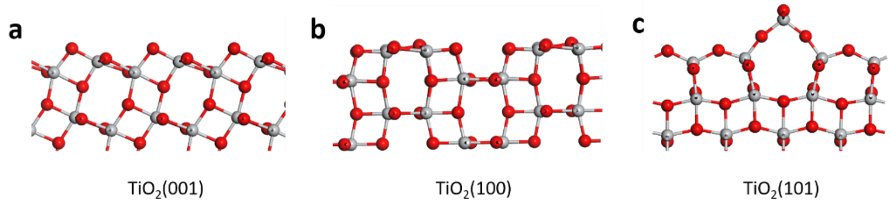

Supplementary Fig. 29. DFT calculations. Optimized structures of (a) anatase TiO_2(001), (b) (100) and (c) (101) surfaces.

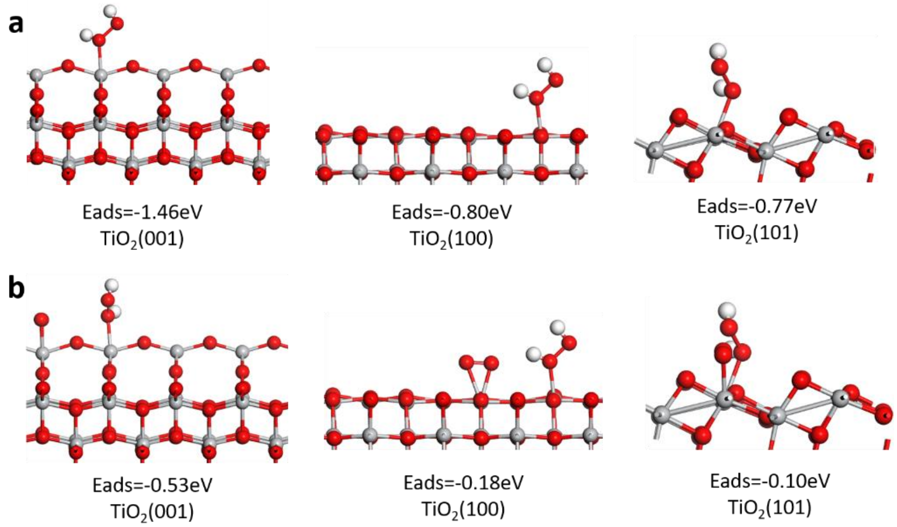

Supplementary Fig. 30. DFT calculations. Optimized structures with corresponding adsorption energies of H_2O_2 adsorption on (a) bare TiO_2 (001), (100) and (101) surfaces and (b) O_2-covered TiO_2 (001), (100) and (101) surfaces.

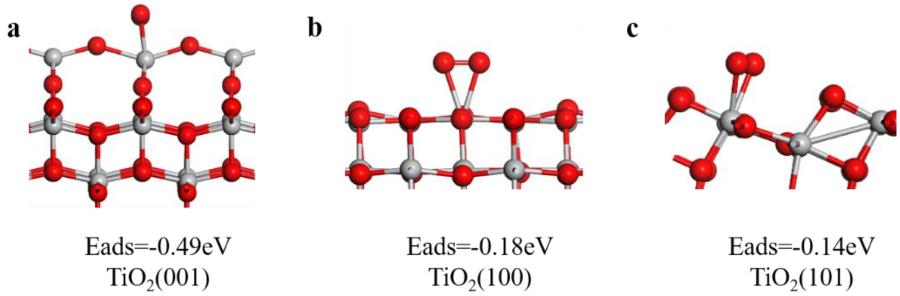

Supplementary Fig. 31. DFT calculations. Optimized structures with corresponding adsorption energies of O_2 adsorption on (a) TiO_2(001), (b) (100) and (c) (101) surfaces.

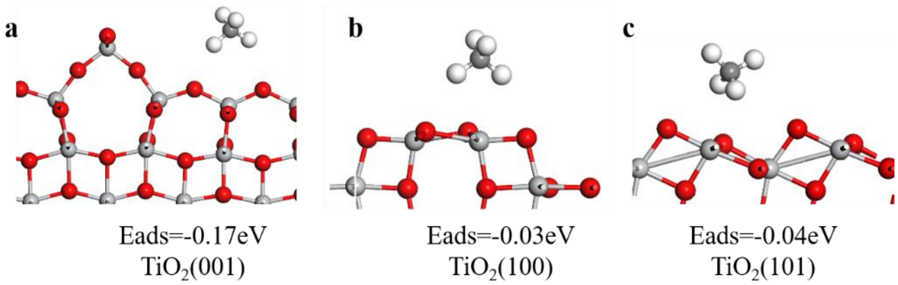

Supplementary Fig. 32. DFT calculations. Optimized structures with corresponding adsorption energies of CH_4 adsorption on (a) TiO_2 (001), (b) (100) and (c) (101) surfaces.

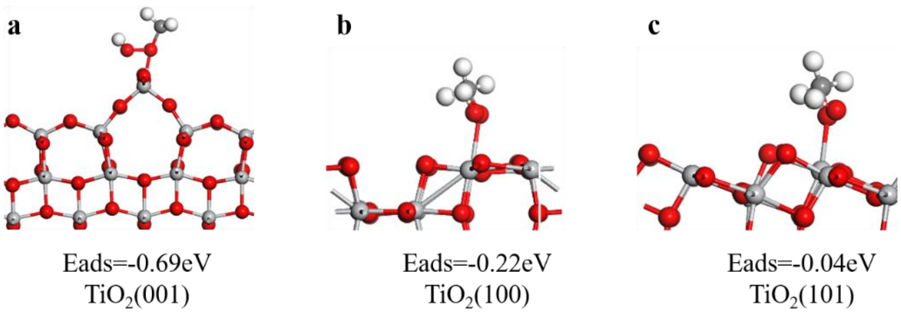

Supplementary Fig. 33. DFT calculations. Optimized structures with corresponding adsorption energies of CH_3OOH adsorption on (a) TiO_2 (001), (b) (100) and (c) (101) surfaces.

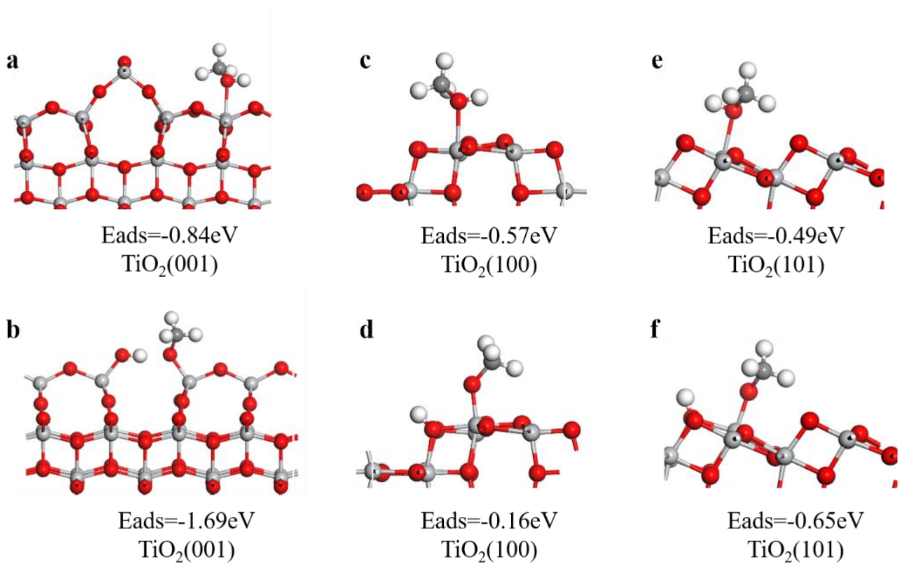

Supplementary Fig. 34. DFT calculations. Optimized structures with corresponding adsorption energies of molecular and dissociative CH_3OH adsorption on (a and b) TiO_2 (001), (c and d) (100) and (e and f) (101) surfaces.

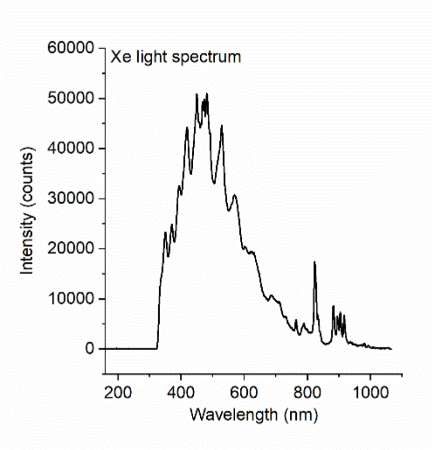

Supplementary Fig. 35. UV light source. Spectrum of the 300 W Xe lamp used as the light source in the work.

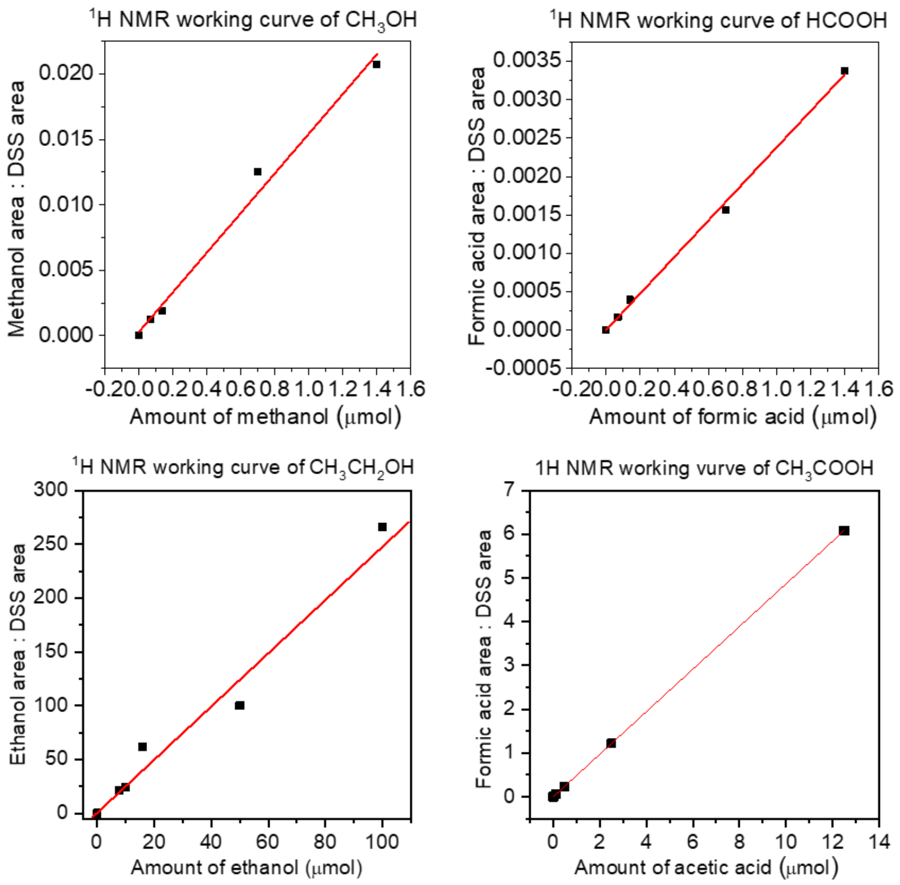

Supplementary Fig. 36. Product analysis method. ^{1}H NMR working curves of liquid-phase oxygenates acquired using pure product of different concentrations. Source data are provided as a Source Data file.

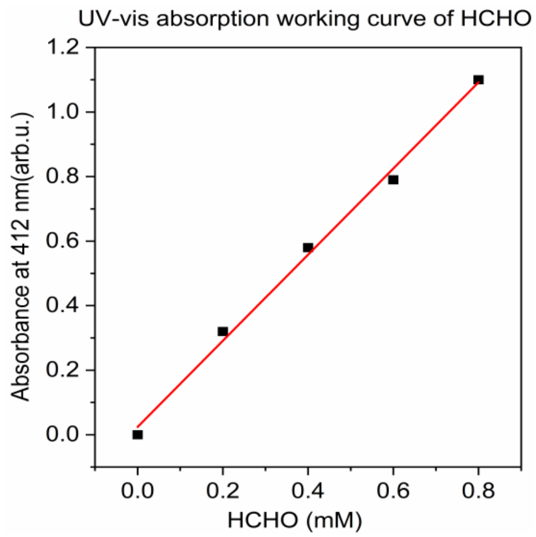

Supplementary Fig. 37. Product analysis method. UV-vis absorption working curve of HCHO aqueous solution. Source data are provided as a Source Data file.

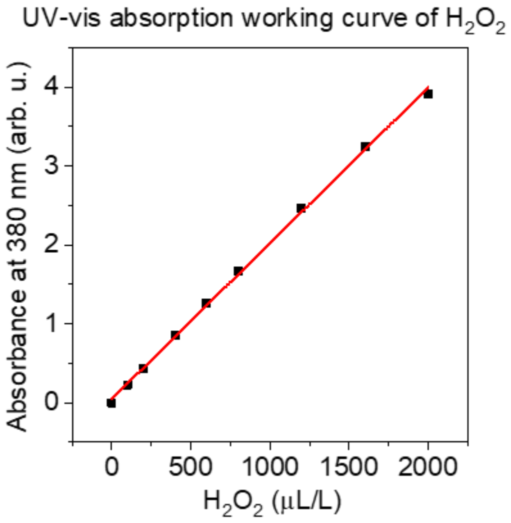

Supplementary Fig. 38. Product analysis method. UV-vis absorption working curve of H_2O_2 aqueous solution. Source data are provided as a Source Data file.

Supplementary References

1. Wang, X., Blechert, S., Antonietti, M. Polymeric graphitic carbon nitride for heterogeneous photocatalysis. *ACS Catal.* **2,** 1596–1606 (2012).

2. Li, H., Shan, C., Pan, B. Fe(III)-doped g-C_3N_4 mediated peroxymonosulfate activation for selective degradation of phenolic compounds via high-valent IronOxo species. *Environ. Sci. Technol.* **52,** 2197−2205 (2018).
